# Navigating the unspoken: A qualitative study of employees’ perceptions of culture and norms surrounding sexual harassment at a Swedish university

**DOI:** 10.1371/journal.pone.0351724

**Published:** 2026-06-16

**Authors:** Jack Palmieri, Frida Pilgaard, Anette Agardh

**Affiliations:** Division of Social Medicine and Global Health, Department of Clinical Sciences, Malmö, Lund University, Malmö, Sweden; Group for Technical Assistance / Asian College for Advance Studies, Purbanchal University, NEPAL

## Abstract

Sexual harassment persists in academic workplaces despite extensive policy frameworks. This study explored how employees at a large Swedish university perceive and articulate the organisational culture and everyday norms that shape sexual harassment. Ten focus group discussions were held with forty staff members, separated by managerial role and conducted in Swedish or English. Transcripts were analysed using qualitative content analysis to identify shared meanings, latent themes and interpretive patterns. Analysis generated one overarching theme, perceiving sexual harassment through the lens of organisational silence, power relations, and negotiated boundaries, supported by four sub-themes. Participants described boundary-setting as a collective, situational process: definitions of harassment shifted in real time, with women often seeking peer confirmation while men framed the same conduct as innocuous. Formal and informal hierarchies amplified this ambiguity: senior researchers with grant-generating prestige were deemed ‘untouchable’, and managers reported uncertainty about how to act without clear procedural guidance. Silence emerged as a strategic response to protect careers and collegial relationships, normalising borderline behaviours through humour and rationalisation. Yet employees also engaged in discrete forms of peer solidarity, staying with vulnerable colleagues after meetings, quietly redirecting collaborations, which signalled a sense of collective responsibility even in the absence of robust institutional support. These findings show that policy compliance alone cannot shift workplace culture when interpretive authority rests with peer groups and incentive structures reward silence. Universities therefore need to focus on organisational level responses that equip leaders with emotional competence and procedural clarity and support the creation of a work environment that can identify, prevent, and respond to sexual harassment. Embedding such measures can transform informal solidarity into a shared, institutionally endorsed standard of respect.

## Introduction

Workplace sexual harassment (SH) remains a deeply embedded problem across countries and sectors worldwide [1]. Since the concept entered academic discourse in the 1970s, research on the prevalence and consequences of SH has expanded substantially [2], with recent meta-analyses by Latcheva (2017) and Debnath (2025) confirming that it remains a serious and under-addressed form of gender-based violence [2,3]. Despite this extensive evidence base, prevention and response continue to fall short, with research suggesting that SH is not only a behavioural problem but also a social and organisational one [2].

One reason SH remains difficult to address is the lack of an agreed upon definition of the phenomenon within the research community. This lack of consensus contributes to complications regarding how SH can be measured and understood [4,5].

At the organisational level, one key factor associated with how SH arises, is perceived, and responded to is organisational climate. Research evidence indicates that a climate that tolerates SH leads to more cases of such harassment as well as lower likelihood of reporting [6]. In addition, SH does not occur in isolation but rather together with other forms of harassment or derogatory treatment, embedded in broader patterns of workplace mistreatment [7]. Together, these findings underscore the need to address not only individual incidents of SH but also the wider organisational norms and relational dynamics that shape how SH is understood and managed in the workplace.

Qualitative studies have further illustrated how organisational norms and social meaning-making influence whether SH is acknowledged, challenged or normalised. At a Jordanian university, SH was formally considered unacceptable, yet more permissive social norms weakened enforcement and discouraged interventions [8]. Focus group discussions among young men in Mumbai similarly demonstrated how gender scripts can legitimise harassment and neutralise validation through peer validation [9]. In European settings, uncertainty regarding definitions, reporting channels, and access to support has been shown to impact reporting and prevention [10], while a qualitative study from Denmark found that employees often struggled to label their experiences as SH, a process shaped by peer responses, cultural discourse, and moments of public reckoning such as the #MeToo social movement [11,12].

Language and discourse can also entrench modes of reasoning that diffuse responsibility and impede accountability. In a discourse analysis at a large U.S. government agency, employees contested the organisation’s SH policy through a set of interwoven oppositions, male/female, emotion/rationality, target/perpetrator, and behaviour/perception [13]. Such binary modalities resonate with narratives from Swedish organisational actors tasked with addressing harassment that emphasise mediation over accountability, potentially reinforcing institutional minimisation and ambiguity [14].

These dynamics are especially central in academic workplaces, where strong hierarchies, dependency relationships, disciplinary silos, and an often-implicit tolerance for informal or boundary-pushing behaviours shape the organisational environment [15]. A 2024 systematic review of gender-based violence in academia found that over half of university staff had experienced some form of interpersonal violence, with women and LGBTQ+ staff reporting disproportionate exposure to SH, stalled career advancement, and reduced psychological well-being [15]. Universities were frequently described as ‘gendered organisations’ in which hegemonic masculinity, competitive norms, and hierarchical control structures inhibit safe reporting and enable cultures of silence, avoidance, and blame [15].

In the Swedish academic context, a cross-sectional survey conducted in 2019 as part of the Tellus project at Lund University reported that 25% of female employees and 7% of male employees had experienced SH during their employment, with corresponding 12-month prevalence estimates of around 8% and 3% respectively [16].

In Sweden, SH is addressed primarily through the Discrimination Act that defines SH as conduct of a sexual nature that violates an individual’s dignity [17]. When behaviours meet criteria for criminal offences, (for example sexual molestation or the capture of illicit images), they are prosecuted under the Penal Code [18]. Harassment that is not sexual in nature can likewise fall under the Discrimination Act, but only if it targets one of the statute’s seven protected characteristics: sex, gender identity or expression, ethnicity, religion or belief, disability, sexual orientation, or age.

Under the Work Environment Act (Arbetsmiljölagen), employers are required to systematically plan, lead, and control their operations to ensure a safe and healthy workplace [19]. In line with the Swedish Work Environment Authority’s regulation (AFS 2015:4), this includes prohibiting victimisation, eliminating conditions that could give rise to harassment, and establishing clear procedures for reporting, investigating, and remedying any incidents [20].

However, existing research highlights how formal policies rarely translate into practice when everyday norms reward silence or trivialise boundary violations [2,11]. It also shows how the social process of naming SH is contingent on collective sense‑making; where organisational narratives that frame incidents as misunderstandings delay or prevent self‑labelling and reporting [21]. Despite this, much of the existing research is quantitative, and the qualitative studies that do exist mostly focus on victims’ perspectives. Employees’ perceptions of the everyday norms and organisational conditions that enable or mitigate SH, including perceptions among those with managerial responsibilities, remain underexplored particularly in academic settings [14]. Understanding these cultural mechanisms is a prerequisite for developing preventive strategies that move beyond symbolic compliance with workplace policies to foster genuinely safe and respectful academic workplaces.

To address these gaps, this qualitative study aimed to explore how employees at a Swedish university perceive the organisational culture and workplace norms surrounding sexual harassment.

## Methods

### Study design

This qualitative study used focus group discussions (FGDs) to gain a deeper understanding of how workplace norms and organisational culture around sexual harassment are perceived and articulated by employees at Lund University. The analytical approach employed was qualitative content analysis (QCA), as described by Graneheim and Lundman [22]. This approach is well-suited to studies seeking to explore variation in perceptions and social meaning-making, allowing for abstraction from manifest content into latent themes.

### Study population and sampling

Data were collected as part of the Tellus project at Lund University. Located in southern Sweden, Lund University employs around 8,600 staff and enrols 46,000 students across nine faculties. Faculty size range from 200 in Fine and Performing Arts to 1,900 in Science, and the proportion of women from 37% in Science to 65% in Law. International employees account for 40% of the workforce, and 35% hold fixed-term contracts. Faculties are headed by deans, and departments by heads of department. Both of these positions are elected [23].

Lund University follows the definition of sexual harassment found in the Discrimination Act, and has a strict zero-tolerance policy to harassment, sexual harassment and victimisation [24].

The Tellus project was a university-wide initiative launched in 2018 to strengthen the prevention of and response to SH among students and staff. Tellus included both qualitative and quantitative components. Key findings from the survey outlining the prevalence and characteristics of SH in this context are presented in an overview article [16].

All staff employed at the university were eligible to participate in this study, regardless of employment type, position, or personal experience of SH. Recruitment to FGDs was carried out from November 14^th^, 2018, to May 7^th^, 2019, and was conducted through posters, digital newsletters, and internal staff communication channels. These posters were displayed on staff notice boards in all faculties and internal communication was sent to all employees via staff email. Those employees who expressed interest in participating in the study contacted the research team via a project-specific email address and received detailed information about the study, including scheduled times and locations for the discussions. All employees who expressed interest in participating were offered a place in an FGD, however, several participants did not attend the FGDs once booked.

### Data collection

A thematic discussion guide was developed (Appendix 1), covering perceptions of SH, situational contexts, forms of expression, workplace dynamics, and expectations for the university’s future response. Although the university has a definition of SH that is based on the Discrimination Act [24], no a priori definition was provided, in order to avoid prejudicing participants’ responses and to explore how SH is understood and discussed among employees. Instead, participants were asked if they believed SH occurred at Lund university, with follow up prompts exploring what this SH looks like, and what forms it could take. The guide was pre-tested with academic staff at Malmö University. Located in southern Sweden, close to Lund University, Malmö University employs around 2,200 staff and enrols 28,000 students across five faculties. International employees make up about 20% of staff [25].

Data collection took place between February and May 2019. In recognition of the influence of formal power dynamics in group interactions, FGDs were stratified by managerial status. Participants who reported having managerial responsibilities were placed in separate groups from those without such roles. All FGDs were audio recorded and transcribed verbatim by a research assistant at the university. The analysis followed the principles of qualitative content analysis according to Graneheim and Lundman [22]. Firstly, all transcripts were read through multiple times to gain familiarity with the data. Following this, meaning units relevant to the research aim were identified and coded inductively in English by the first author. These codes were then grouped into sub-categories and categories through a combination of inductive and deductive processes, moving iteratively between the empirical data and emerging interpretations.

Categories were subsequently examined for the development of latent sub-themes through a process of pattern recognition and conceptual reflection. Finally, one overarching theme was formulated. Quotes were selected to illustrate the categories, and these were translated to English by the first author. Data analysis was conducted using OpenCode 4 [26]. The final model was developed collaboratively, with all co-authors reviewing coding decisions and contributing to analytical refinement.

### Ethical considerations

Ethical approval for the study was granted by the Swedish Ethical Review Authority (reference number: 2018/350). All participants were provided with detailed written and oral information prior to the FGDs, outlining the purpose of the research, the structure of the FGDs, and the voluntary nature of participation. Individuals were informed of their right to ask questions and seek clarification before providing written informed consent.

Given the group format of the discussions, participants were explicitly informed of the limitations to confidentiality. While the research team could ensure secure handling and storage of data, confidentiality among group members could not be guaranteed. To support open dialogue and minimise potential power imbalances, participants with managerial or supervisory responsibilities were assigned to separate FGDs from those without such roles, thereby avoiding situations in which staff and their line managers would be placed in the same discussion.

During the FGDs, participants were given the option to use their real names or a pseudonym. All were encouraged to maintain the confidentiality of what was shared within the group. At the end of each session, participants received an information sheet listing relevant organisations and services available for psychological or emotional support, in case any distress arose during or after the discussions.

All identifying information was removed during the transcription process to protect participant anonymity. All audio recordings were transferred to encrypted hard drives immediately after the FGD and deleted from the audio recorders. These audio recordings and transcripts were securely stored on encrypted drives with access restricted to members of the research team. Consent forms were stored separately in a locked safe.

## Results

A total of 40 individuals participated in 10 FGDs, five with participants in managerial roles and five without. Participants were offered a choice of attending FGDs in Swedish or in English. Group sizes ranged from 2 to 7 participants (average 4), and discussion lengths ranged from 53 to 84 minutes (average 67 minutes). All FGDs were held in university-owned rooms. Six of the group discussions were moderated by the first author (JP), with the last author (AA) as co-moderator, and four FGDs were moderated by the last author (AA) with the first author (JP) as co-moderator. A seating plan showing the participants (by number and sex) was produced for each FGD, and a diary with notes made both during and immediately after the FGDs was kept ensuring a clear audit trail. An overview of participant characteristics is presented in Table 1.

**Table 1:** Focus group participant characteristics.

FGD#	# Participants	#Female	#Male	Managerial responsibility	Language
1	6	2	4	Yes	Swedish
2	3	0	3	Yes	Swedish
3	4	3	1	No	Swedish
4	4	4	0	No	Swedish
5	4	2	2	No	Swedish
6	3	3	0	Yes	Swedish
7	4	1	3	Yes	English
8	2	2	0	No	Swedish
9	7	3	4	Yes	Swedish
10	4	4	0	No	Swedish

The analysis of the focus group discussions resulted in one overarching theme, four sub-themes, and eight categories that reflect how Swedish university employees perceive the organisational norms and workplace culture surrounding SH. The findings offer insight into how participants interpret, negotiate, and respond to the structural, social, and cultural dimensions of harassment within their professional environment. Rather than focusing solely on personal experiences, the analysis highlights how shared understandings, implicit expectations, and institutional responses shape perceptions of what is acceptable, what is silenced, and what can be challenged.

The overarching theme, Perceiving sexual harassment through the lens of organisational silence, power relations and negotiated boundaries, was supported by four sub-themes and eight categories. The sub-themes were: Negotiating collective norms and meanings, Navigating power relations and organisational management, Facing a culture of silence and normalisation, and Offering peer support for collective action. Each sub-theme is created from categories that reflect how harassment is not only a matter of individual behaviour, but deeply embedded in the organisational climate, power relations, and everyday interactions.

**Fig 1:**
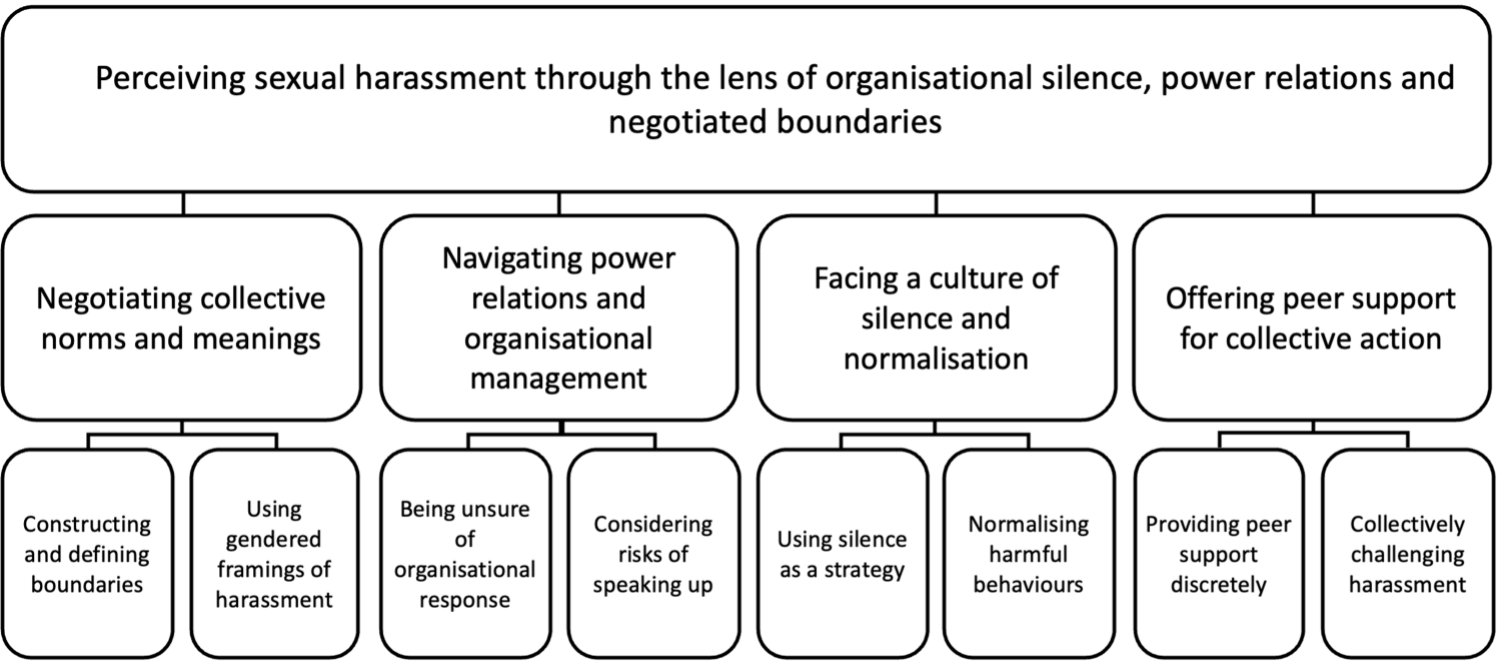
Overview of the main findings of the study: categories, sub-themes, and overarching theme.

**Fig 1** presents an overview of the results, outlining the overarching theme, sub-themes, and categories that emerged from the analysis.

In the following paragraphs, results are presented in greater detail. The overarching theme and sub-themes are shown in **bold**, and categories in ***bold italics***. Quotations and excerpts from the FGDs have been included to demonstrate how interpretations are grounded in participants’ language and situated dialogue.

### Overarching theme: Perceiving sexual harassment through the lens of organisational silence, power relations, and negotiated boundaries

The overarching theme reflects how participants made sense of SH not simply as individual acts of misconduct but as phenomena embedded in the fabric of university life, shaped by institutional cultures, informal norms, and relational hierarchies. Perceptions of what constitutes SH were contingent on dynamic and negotiated boundaries, often shaped by peer interaction, managerial response, and broader societal shifts such as #MeToo. Across the discussions, participants highlighted how unclear definitions and what they experienced as changing expectations created ambiguity, fostering environments where boundary-crossing behaviour was normalised or explained away. Power relations, both formal and informal, were seen as key to understanding who was protected, who was heard, and who bore the risks of speaking up. Silence emerged not only as a symptom of organisational failure but as a protective strategy, adopted by employees navigating uncertain terrain. As a result, participants described environments where misconduct could flourish, not due to ignorance, but rather because of varying interpretations of situations, tacit tolerance, reputational concerns, and the absence of consistent institutional accountability. Taken together, these dynamics reveal how harassment was perceived through a cultural lens, one in which silence, power, and contested norms continuously shaped the boundaries of what could be said, challenged, or changed.

### Negotiating collective norms and meanings

This sub-theme focuses on how participants collectively shaped, contested, and reinforced understandings of SH. It shows how, in the absence of clearly defined definitions, understandings, and boundaries are actively negotiated. Throughout the discussions, participants contested boundaries between appropriate and inappropriate behaviour, interpreted incidents through cultural and gendered lenses, and expressed evolving understandings over time. This sub-theme is supported by two categories.

#### *Constructing and defining boundaries*.

Participants described challenges in establishing clear definitions and shared understandings of SH in the university setting. Participants engaged in the creation of collective narratives through which they together built an understanding of SH.

*M1 “But how would you distinguish that? You can’t always say it’s a form of power; there are so many informal power structures.”*

*M3 “There isn’t a universal definition, and there isn’t a one-size-[fits-all] either. In each and every case, you need to define the boundaries. What classifies as this and what doesn’t. The context matters.”*

*F2 “It can be body language too. It doesn’t even have to be words.”*

*M1 “Exactly. And I think it’s important to remember, the target has the right to interpret this first.” (FGD 7)*

In most of the focus group discussions, this collective defining resulted in definitions that revolved around the primacy of the victim to interpret the situation they have experienced as SH or not. At times, however, the discussion moved away from defining what SH was and instead trusting in the role of the university to define ‘acceptable behaviour’ and any deviations that could be harassment.

*F2 “At least in my experience, it’s not the obvious stuff. It’s more subtle. People are numb to it, and they don’t know they’re doing it. It’s just part of their behaviour.”*

*F2 “Exactly. They’ve just never been confronted.”*

*M3 “It’s not always for sexual gratification either. It’s about power. Sexual harassment is one form, but others use intimidation or exclusion.”*

*M1 “You can’t draw a clear line. Is a dirty joke harassment? A picture on a whiteboard?”*

*M3 “It’s about how we define acceptable behaviour, institutionally.” (FGD 7)*

Participants also discussed how the lack of a clear definition directly impacted their ability to talk about SH, but also to act and react when there is no common understanding or tools to help understand this in the university setting.

*“And that the question might also be asked… ‘what could it be?’, to students and staff. So that there is something to start from. That way it becomes easier to identify if something like that were to happen. Or to know how not to behave in a certain situation. Or to create a tool that makes it easier for someone who is unsure to ask, ‘is what I’m doing now sexual harassment?’ To create some kind of tool that allows one to even ask the question, ‘do you feel uncomfortable when I do this?’ or ‘does this feel weird?’ or ‘how do you experience this now?’ Because those tools are missing, at least that’s my experience.” (FGD 1, F)*

In the context of how SH was understood at the university, participants discussed the question of students and staff from settings outside of Sweden. Concerns were raised that those with different backgrounds might not only have a different framework for action, but also that they might interpret situations of harassment differently.

*“Okay… do they understand this? Do they understand their rights? Do they understand what is allowed and not allowed?”. And you know they’re coming some of them from different countries. From different cultures and so on, so on.” (FGD 7, M)*

These discussions tended to result in participants asking for specific interventions to support international staff, or to ‘protect’ other staff from this group. One such intervention was the production of a code of conduct to regulate what behaviours should be considered acceptable or not, and that would apply across all settings.

While definitions and the norms surrounding them were clearly contested, there was a general feeling that #MeToo had played a significant role in changing the climate for discussion. This ranged from a hope that #MeToo would make people *“more likely to speak up” (FGD 8, F)*, all the way up to seeing #MeToo as a *“wake-up call” (FGD 9, M)* that had reduced the taboo around the topic of SH and paved the way towards allowing people to identify situations as harassing and feel supported in saying no.

*“Previously, before MeToo, definitely ten years ago, people thought, ‘Well, you just have to put up with that if you’re pretty,’ or ‘You get that because you’re so damn nice.’ Now people are more like, ‘Okay, maybe that’s not okay.” (FGD 9, F)*

Other participants were less positive about MeToo, however, worrying that the movement was primarily *“rhetoric” (FGD 10, F)* and had hidden a lack of progress in addressing SH. Others went even further, discussing how they actively *“disliked”* the movement due to what they see as its role in conflating serious and less-serious issues and, in doing so, muddling the boundaries of what is acceptable *(FGD 7, M)*.

#### *Using gendered framings of harassment*.

At times in the discussions, gender differences in the way that participants framed the issue of SH became apparent. This included both the type of language used when discussing examples of SH, as well as differences in interpreting situations as SH, and understanding the consequences of such harassment.

In terms of how the topics were framed, male respondents frequently used institutional, procedural, or philosophical terms to describe SH, sometimes distancing themselves from the emotional or experiential aspects. This also had the consequence of flattening or generalising distinctions that could be seen as a dilution of the specificity of SH.

*“Maybe it’s not really that interesting where the boundary is, whether it’s sexual harassment or another kind. In the end, it’s all harassment really.”(FGD 9, M)*

Male participants were also more likely than their female equivalents to cast doubt on SH or draw boundaries between harassment and “innocent” or “unintended” actions.

Female participants, on the other hand tended to use personal, affective, and specific language, that often emphasised a large degree of interpersonal dynamics.

*“It’s always the one who experiences it that counts. I might not have meant anything by it, but if someone else feels harmed, then that’s what matters.” (FGD7, F)*

The differences between genders went beyond the language and framing used, however, to include instances where male and female participants were unable to agree upon whether a situation constituted SH or not. Showing clear undertones of gendered language, the participants were unable to understand each other’s lived experiences or agree upon whether the act or the situation is important. In one example, a male participant argued that the victim of the alleged SH was himself vulnerable because he was male, while for the female participant it was the act of SH that was important.

*F2 “There’s no difference. It’s unwanted attention.”*

*M3 “Yes, but in the broader perspective, one person is vulnerable because of their identity and the other because of an act committed against them.”*

*F2:“Yes, but in terms of sexual harassment, there’s no difference.”*

*M3:“No, there isn’t. But those different aspects come into play.”*

*F2:“Mm, but it’s the act that matters” (FGD 5)*

Female participants also reflected over actions and language used by male colleagues that had made them feel extremely uncomfortable, but where the perpetrator had not at all understood the seriousness and consequences of their words.

*“…he said, about her, something like ‘she’s damn hot, but with an ass like that maybe she shouldn’t be wearing that skirt’. He said it because he thought we had that kind of relationship, God knows why, but she didn’t hear it, so it didn’t affect her directly. At the time, I just told him I didn’t appreciate that kind of comment, but I didn’t do anything more. But it was such a strange… A strange thing to say, and what was so frightening was the ease with which he said it, as if he might just as well have said ‘the meatballs were good at lunch’.” (FGD 4, F)*.

These differences in gendered framings of SH contributed to the more general problem of defining what SH is in a university setting. This in turn led to a lack of common language that made it difficult to discuss the phenomenon.

### Navigating power relations and organisational management

This sub-theme described how employees at the university are critical of some elements of organisational structures and uncertain about how leadership might respond to SH claims. This uncertainty led to a concern that reported incidents were not treated fairly or consistently. Fears of consequences to speaking up had an impact on decisions to react or report SH, and this was true for both consequences for one’s career and their social situation.

#### *Being unsure of organisational response*.

Participants discussed how power and dependency relationships that exist within the university structure shaped both feelings of responsibility and risk. The siloed system of the university was raised as contributing to a large variation of experiences across the organisation. This variation included both how SH could occur, how it would be handled, and what systems and routines were in place to support this. As one participant put it

*“We work in our own bubbles. It’s not like you know what’s happening at other departments. Even within the same faculty, it’s like small islands.” (FGD 9, F)*

This isolation and variation was exacerbated by the perceived role of individuals at the different department especially the power of the head of the department (prefect).

*“We’re entirely dependent on the head of department. And if you’re unlucky, that’s it. There’s no system that lets you go above someone.” (FGD 5, F)*

In some cases, negative experiences with leadership were described, with heads of department exhibiting an *‘Ostrich effect’* whereby they ‘bury their heads in the sand’ and ignore any information or issues that might cause them discomfort (FGD 7, M).

The powerful position of department heads was exacerbated by a perceived lack of training and competence in handling sensitive topics. Those in leadership positions were seen as requiring support and training, with professionalism and *“emotional intelligence” (FGD 7)* as key elements.

*“…today it’s enough to have money to become a research group leader, but that doesn’t mean you’re suited to be a leader. What does that responsibility actually entail? I think what we really need is a more professional leadership culture that helps us move away from the old hierarchies and toward an understanding of what leadership actually requires.” (FGD 8, F)*

In addition to struggling to understand the formal structures at the university, participants spoke of power dynamics hidden in informal relations. These hierarchies could be related to who has research funding, or who has been employed the longest, and were seen as being hidden by the idea of flat hierarchies and the perceived security due to societal stability referred to by one of the participants as ‘Scandinavian complacency’ (FGD 7, M). The result of these hierarchies were that *“some people seem untouchable, like no one dares to question them.” (FGD 6, F)*.

*F2 “When the person doing it is very senior, like a professor who’s been here forever, it’s like nobody dares say anything. They just lower their heads and try to work around it.”*

*M3 “Exactly. And if they bring in a lot of research money, it’s even worse. Then it’s like, ‘just don’t rock the boat.’” (FGD 5)*

As a consequence of the perceived arbitrary nature of power in the university system, participants discussed how they felt they had to anticipate leadership reactions, and that this in turn led them to make decisions about whether to act or not. Rather than expecting proactive or strategic engagement, many participants assumed that institutional responses would be reactive or performative, sometimes driven by public pressure rather than organisational reflection.

*“That worries me when a structure or an organisation is doing something because it’s jumping on a bandwagon (…) Sexual harassment is a manifestation of a problem (...) it’s a consequence of something else (…) and that’s the whole work culture.” (FGD 7, M)*.

This perception contributed to widespread *questioning of fairness and consistency*. Participants voiced concerns about unclear procedures and a lack of transparency, worrying that the rules might be unevenly applied when *“issues are handled behind closed doors” (FGD 1, F)*. This also resulted into a discussion about whether there were any ‘good examples’ of perpetrators facing consequences – something that contributed to the feeling of a lack of fairness and consistency.

*M1 “But do we have those stories about what happened to the perpetrator? When the punishment came? That is hard to find, a good end to that one.”*

*F2 “Very.”*

*M1 “A good end to that one.(…) ‘this is what happened, this person did this to this one, and then they reported it, and this person is now not working with Lund University anymore.’”*

*F2 “Exactly. That’s all you need.”*

*M1 “Or whatever (laughs). Something that could feel fair,” (FGD 7)*

Across the FGDs there were notable differences in the type of language used by those in leadership roles and those without formal responsibilities.

Those with leadership or managerial positions tended to **frame the importance of structural responsibility and to emphasise systemic change. This could be seen through the ways in which they** often reflected on institutional deficiencies, staff training, and leadership within a broader organisational lens, and with any response needing to *“Penetrate everything, budgeting, hiring, teaching.” (FGD 7, M)*.

At the same time, these leaders also expressed uncertainty about their role and competencies, discussing how they have to walk the line between being supportive and following the rules.

*“I’ve had difficult situations in my team. [...] I’m not a lawyer. What I see is a complete lack of support. We don’t even have a handbook for this.” (FGD 7, M)*

There was also frustration with how the system currently collects information, with those in managerial positions torn between action and inaction. This was compounded by a perception among the managers and leaders that you could not respond without an official report.

*M6: “At the same time, it’s important to report.”*

*F1: “Yes, absolutely.”*

*M6: “As a manager and leader, it becomes incredibly difficult if no one dares to come forward. You hear rumours, and there’s whispering and secrecy and so on… but if no one can give a concrete example and dare to say, “my experience” or “I know,” then it’s simply not possible to address it properly either. I think there’s a large dark figure. I think there’s a huge dark figure.” (FGD 1)*

At the same time, leaders also expressed being stuck between a desire to support colleagues in the way they wanted, and the formal responsibility they had to report, and the need for an investigation to allow this.

*M1 “You can always file an incident report; there you can be anonymous.”*

*F3 “…but if you need to escalate it, you have to come forward as a person.” (FGD 3)*

Those without leadership or managerial responsibilities focused more on lived risk, silence, and uncertainty as well as observed inaction from the leadership. Their concerns were with the nature of recruitment of department heads, and their perception that so much of the response depended on the individual characteristics of these managers**.**

#### *Considering risks of speaking up*.

A pervasive sense of risk was apparent in the focus group discussions with participants fearing social or career-related consequences should they report what had happened. These risks were often described as being a consequence of the hierarchical nature of the university system, as well as the relationships of dependency reflected in both formal and informal power dynamics. The consequences described were broad ranging, and several participants spoke of fears about their career should they report.

*“It’s this again, this issue of hierarchy and dependent employment, what are the consequences if you speak up? What kind of relationship is it, what’s the age difference, what factors are involved, and if it’s a successful, prominent researcher (…) then maybe you don’t dare to speak up because it could ruin your entire career.” (FGD 8)*

Others were concerned about more insidious consequences such as being left out or labelled as the ‘problem’.

*M1 “They don’t believe anything will happen. They have seen other cases where they become the problem*.

*M2 “Disruptive, yeah.”*

*M1 “How are we going to contain this [...] or move this person that is always complaining? Even if the prefect doesn’t remember it, it’s still this ‘problematic woman’ who once complained. And I’ve heard that so many times.”*

*F1 “Tribal knowledge, I call it. (F)” (FGD 7)*

These consequences were not seen as exclusively remaining at their workplace, however, but included more social ‘punishments’ such as exclusion from office events during the evenings and weekends.

In a small number of cases, participants discussed how fear of being accused of some sort of harassment or SH had led to male colleagues avoiding putting themselves in situations where they were alone or ‘social’ with female colleagues. This in turn had led to these female colleagues feeling excluded and treated differently.

*“Does it count as sexual harassment if you feel excluded because your colleagues or supervisors are afraid to include you, since they’re afraid of being accused of sexual harassment? Because I’ve experienced that, as a female doctoral student, I was treated differently than the male doctoral students by certain supervisors who wouldn’t have a beer with me like they would with the guys, because they were so afraid of all this.”(FGD 10, F)*

### Facing a culture of silence and normalisation

When describing the discussion climate at the university, participants spoke of a culture of silence, as well as a perceived normalisation of SH. Rather than viewing silence as mere passivity, however, participants framed it as a deliberate strategy, one shaped by fear of repercussions, hierarchical dependencies, and the perceived futility of reporting. At the same time, the discussions highlighted how inappropriate conduct becomes routinised through everyday interactions, humour, and institutional complacency. Participants described how behaviours such as sexist jokes, boundary-crossing, and dismissive comments are often downplayed, trivialised, or explained away as cultural, generational, or accidental. These dynamics contribute to an organisational culture where speaking out is discouraged and misconduct is rendered invisible.

#### *Using silence as a strategy*.

Participants spoke about silence as a response to SH at the university. For some this was due to internalised guilt and self-blame that led to participants avoiding official procedures. For others fear of the consequences of speaking out led to silence.

*“People are probably afraid, it turns into a culture of silence… and I don’t think we can get around that (…) you feel like it’ll just come back on me…” (FGD 3, F)*

This decision was further exacerbated by relations of dependency and power dynamics between victims and perpetrators in the university system.

*As a PhD student, you don’t report the guy supervising your thesis. Even if something happens. You just try to get through it, because you’re dependent on them for everything, your career, your funding, your future.” (FGD 9, F)*

Thus, remaining silent was seen as a strategic decision for some. Employees knowingly dealt with the potential consequences of their harassment and not reporting to protect their own position. This resulted in people adopting silence as a strategy and *“(learning) what you can say and what you shouldn’t” (FGD 1, F)*. Employees also openly discussed how they kept their heads down to get on with the job and to ensure that the situation did not escalate. This even extended to the decision not to report witnessed harassment. Protecting the victim and ensuring things did not escalate were given as reasons for not reporting

*“If you, if you report something like this… then you have to be aware of the responsibility you take on in relation to, also to the victim. Who might… be in a situation where that person… doesn’t dare to move forward, or something like that. And then, there’s always a risk that you cause more harm” (FGD 1, F)*

#### *Normalising harmful behaviours*.

Participants in the focus group discussions detailed how harmful behaviours and SH at the university were downplayed, trivialised and ‘explained’ away. These reactions, conscious or not, were seen as contributing to the normalisation of SH at the university, as well as closing down the potential space for reporting.

In terms of downplaying SH, the focus group discussions include examples of both minimising other’s experiences as a way of downplaying the problems and denying their own experience as being serious.

*“Although they (the colleague) felt it was very inappropriate, for me it was nothing, it was really something light” (FGD7, M)*

This also took the form of denying that certain forms of behaviour should be considered harassment at all and limiting the definition to more explicit forms only.

*“That can be hard… I mean, it’s not always clear. It’s not like someone is touching you or saying something crude.” (FGD 2, M)*

Among the discussions were also clear examples of where colleagues to the participants in the discussion had seen humour used to trivialise events and, in doing so, legitimise certain actions.

*M1 “Yeah, maybe the worst. That there are structures that normalise this behaviour… it becomes allowed, it becomes the joke, the sexist jokes… become non-events.”*

*F2 “And I know a few male colleagues who just smirk, like, and act like it’s silly or dumbcane that’s where I think group discussions would help. Just hearing how others think and talk about it, that could actually make quite a difference.” (FGD7)*

Participants also normalised SH themselves through explaining certain events away. This was done through highlighting differences in how the older generation understands harassment, exploring whether cultural differences in how SH is understood can explain its occurrence, or through framing SH as unintentional and thus less serious. These three perspectives are represented in the quotes below.

*“…it was again one of those senior men who thinks he’s in love and can’t manage a private relationship at work… they’re not vile in the same way, just old men being silly.”(FGD 6, F)*

*F2 “What we perceive as… let’s just call it harassment in general, has a lot of different levels. Depending on your culture, where you came from, your background… a myriad of things.” (FGD7)*

*“I don’t think it’s always intentional, like it’s not as if someone planned to do something inappropriate.” (FGD 1, M)*

The combination of a normalisation of SH and a culture of silence was seen to be both a consequence of the power dynamics embedded in the formal and informal structures of the university, and also a factor in the continued occurrence of SH, and the difficulties the university faced in resolving this problem.

### Offering peer support for collective action

The final sub-theme captures how participants saw employees pushing back against the structures and silence at the university. Resistance was expressed through solidarity, trust-building, and emotional courage. These acts of care helped some participants transform their work environments, even when formal mechanisms were weak, and led to norms concerning solidarity and collective support.

#### *Providing peer support discretely*.

Despite organisational limitations, some participants described efforts and experiences of providing support to one another often discretely and through informal means. Colleagues were seen as the most trustworthy partner to turn to, and peer support was seen as vital in order to continue to work if something has happened in your workplace. Peer support was often informal but sometimes intersected with the formal structures such as support in navigating the university systems.

*“Luckily, I had other male colleagues I could tell about this, and they helped me navigate things in the research context, without it getting difficult. Like, I could say, ‘I’m not comfortable, could you handle this conversation?’ or ‘this has happened, so you should know that our collaboration isn’t working anymore’, and so on…” (FGD10, F)*

At other times peer support was more about feeling reassured that you were not to blame and getting confirmation that what you had experienced was serious.

Despite the clear benefits espoused of seeking peer support, turning to peers did not always meet the expectations of the victim. In one case, the peer in question advised the participant not to respond to the offensive act so as to avoid the situation escalating.

*“I also talked to another professor who unfortunately is of the other gender and, because he’s older, I thought ‘okay, maybe he can,’ you know, ‘we can agree at least’, and he said, ‘but you, don’t go there’, like, ‘shut up, stay out of it’, ‘don’t get involved’. So that’s what he said to me, this more experienced colleague.’ (FGD 9, F)*

#### *Collectively challenging harassment*.

Despite a reluctance to get involved with the case of others, either for fear of negative consequences for oneself or for them, participants framed responsibility for dealing with SH as both top-down and bottom-up.

*F2 “We can’t hide behind being a university and say we have knowledge. We have to start thinking differently. Responsibility has to go from the top all the way down so that employees can feel safe, that if something happens, they can report it, and someone will take it seriously.”*

*F1 “Exactly. You need to create trust. The university has to show that this is taken seriously in a professional way. Everyone, from leadership to the rest of us, has a responsibility.” (FGD 8)*

Participants also expressed how they consider the consequences for others when making the decision to report or not. This led to feelings of accountability for collective work environment. If they did not report these issues, then they could be responsible for the consequences for colleagues who might be exposed to similar harassment.

*F2 “(…) by not reporting, by not reacting, you’re exposing others to it. You have to weigh that, ‘Is this about me, or about the person doing this?’ You’re responsible for others too.”*

*M3 “There are informal channels,”*

*K2 “But not doing anything still means you’re taking on responsibility for what might happen to someone else.” (FGD 5)*

These discussions were also marked by an awareness of power, with participants speaking of using their own position of power to support those without.

*F3 “It’s a difficult situation... but I feel responsible for the student workers. They’re like my kids in a way, so if someone acted inappropriately, I’d feel a strong instinct to protect them.”*

*F2 “Exactly. If someone is being harassed, that means they’re in a weaker position, and then I absolutely want to stand up for them, no matter what. I’ve done that for my students. They know I’ll back them up, and I hope that’s valuable to them.”*

*F1 “Even if it hasn’t been about harassment, just unfair treatment, I’ve told people: ‘You have to speak up about this. This isn’t okay.’ And if they say, ‘But you have a permanent position, can’t you say something instead?’ That tells you a lot. There’s a lot of fear among people on temporary contracts.” (FGD 6)*

The perceived need for collective responsibility was also clear from the ways in which bystander roles were discussed by the participants. Participants expressed a growing readiness to act, although ambiguity about when and how to intervene persisted.

*F2 “Today I’m absolutely sure I would do something, but 15 years ago, I’m not sure. But now, I would say something, no matter who it was.”*

*F1 “But I’d need to know that it was unwelcome. That it really was harassment.”*

*F3 “Exactly! (laughs) I mean, if I’m walking past and I just see two people, I don’t know them, it’s hard to know. If it’s violence, it’s different. But if someone’s touching someone, maybe they’re a couple? But if I saw a colleague doing that to someone, yeah, I’d think that was really weird.” (FGD 6)*

## Discussion

The findings of this study illustrate how employees at a Swedish university understand sexual harassment (SH) as a socially embedded phenomenon, perceived through the lens of organisational silence, power relations and negotiated boundaries. Participants discussed how understanding SH was a collective process of constructing and defining norms, and that the boundaries between SH and other forms of harassment were fluid. Power dynamics, both formal and informal, permeated every aspect of the occurrence and handling of SH, and the unique structures and processes inherent in the university system exacerbated this. Employees also discussed how, despite improvements since the #MeToo era, there is a widespread culture of silence and normalisation in their workplace. This silence was seen as both a systemic failure, but also a protective strategy adopted by the employees to protect themselves and their careers. Finally, participants discussed how much of the support currently provided to them comes from colleagues, and that this is often done discretely. Employees felt a level of collective responsibility for the work environment and expressed that challenging SH should also be a collective action.

### Negotiating collective norms and meanings

Lund University is a large institution with a complex management structure and a wide array of work environments, each governed by its own set of rules, regulations, and workplace cultures. Despite this diversity, the University applies a single, consistent definition of sexual harassment, drawn directly from the Discrimination Act, to all contexts and units across the organisation [24]. It is in this context that participants in this study struggled to define what constitutes sexual harassment. Throughout the discussions the definitions were constantly changing, and grey zones were identified. Seeing the boundary of what constitutes SH as a negotiated reality, mirrors what classic symbolic interactionist theory (coined by Herbert Blumer in 1937 [27]) terms a negotiated order, an interpersonal process in which actors continuously redefine situations through talk, eye-contact and tacit cues [28]. Understanding that the boundaries of what constitutes SH are negotiated through interpersonal processes has consequences for the design of preventative policies. By moving the locus from individuals to groups, policies must focus on organisational level-responses and support the need for ongoing dialogue and layered training provided to all at the workplace.

This struggle to define SH is not surprising given that research has found that both individual and organisational characteristics, as well as broader societal norms and values, shape how behaviours are interpreted as SH [29]. For instance, research has shown how women identify a wider array of actions as harassing than men do, and perceptions shift depending on the sex of both perpetrator and victim [30,31]. Likewise, Otterbach, Sousa-Poza and Zhang found that even in countries with high gender egalitarianism, men consistently report lower levels of perceived harassment than women, suggesting that cultural endorsement of equality narrows but does not eliminate these gender gaps in recognition and reporting [32].

Perceptions of what constitutes SH are also influenced by the disjuncture that can occur between legal/regulated definitions of SH and everyday experiences. Statutory definitions, often confined to quid pro quo or overt ‘hostile environment’ criteria, routinely overlook other forms of SH, leaving many harmful behaviours outside the scope of what victims, bystanders, and even decision-makers recognise as sanctionable [33]. Our findings mirror this disconnect, highlighting the gap between formal definitions and lived experience.

Cultural differences likewise shape perceptions in ways that can impede a shared understanding. A meta-analysis of U.S. studies conducted by Ilies et al. in 2003 reveals substantial variation in reported harassment rates across industries and demographic groups, suggesting that national norms, workplace cultures and individual backgrounds influence whether similar behaviours are labelled as harassment [34]. In a multicultural institution such as Lund University, these findings underscore the imperative for policies and training programmes that not only reflect legal requirements but also explicitly address cultural and contextual factors.

In the absence of a common understanding, labelling events as SH becomes more complicated, possibly resulting in fewer cases being reported and thus being resolved. This hesitancy to label incidents as SH is echoed in findings of a qualitative interview study conducted across five European countries including Sweden. In this study by Hagerlid et al., three factors were identified that acted as obstacles to identifying SH namely preconceived notions, blurred boundaries, and competing interpretations [35], all findings confirmed in this study.

Many participants explicitly requested a clear definition of sexual harassment during the focus group discussions, underlining the importance of a shared framework for recognition and reporting. This ambiguity, especially around non-physical behaviours, is reflected in a large cross-sectional study among public-sector employees in Denmark that found as many as 75% of ambiguous behaviours were unlabelled as harassment [11]. Moreover, our findings show that participants, particularly women, often ‘tested the water’ with a trusted peer before deciding whether to label an incident as SH, a reliance on bystander validation similarly documented in low- and middle-income countries [36].

The gendered nature interpreting scenarios and deciding whether, or how, to respond has also been measured quantitatively in large US surveys, where male respondents classified fewer acts as harassment than their female counterparts and attributed shared responsibility to the (female) victims [37,38]. These findings are reflected in our results where male respondents tended to question cases of SH more often as well as label more instances as benign. Men’s tendency to underestimate behaviours that amount to sexual harassment, a form of misconduct that disproportionately harms women, is compounded by their persistent overrepresentation in leadership roles. National data from Statistics Sweden show that men occupy a significantly higher share of managerial positions than would be expected from the overall gender distribution in both the public and private sectors, a pattern that has changed little in recent years [39]. At Lund University, the 2019 equality report reveals that women hold just 28% of professorships and 41% of senior lectureships [40]. These entrenched imbalances at the top risk perpetuating the ‘yellow zone’ of ambiguous misconduct, as male-dominated decision-making bodies may be less attuned to recognising, addressing, and formally sanctioning inappropriate behaviour.

The collective nature of establishing boundaries was seen to have been both aided and hampered by the #MeToo campaign. Participants insisted that the movement had in some ways expanded the space for dialogue yet simultaneously ‘muddied’ the scale of severity, producing confusion about what is ‘serious enough’ to report. There is some sociological evidence, primarily from the USA, that the #MeToo era has contributed to more reports of SH by females being taken seriously [41], which would broadly correspond to the findings in this study. At the same time, a large study conducted in Norway and utilising a web-based questionnaire found that negative beliefs about the #MeToo movement, such as fear of false accusations overshadowed the effects of positive beliefs about the outcomes, for both male and female respondents [42].

The fluid, peer-centred boundary-setting we observed, where employees ‘sense-check’ ambiguous incidents with colleagues before deciding whether to label them harassment, echoes a wider pattern in which social groups use shared norms to legitimise or downplay troubling behaviour. This process of collectively negotiated meaning‑making echoes findings from studies from South African Universities, where focus group‑based research among students and staff has shown how everyday verbal and physical violations are frequently framed as ordinary expressions of masculinity or institutional life (“dangerous common sense”), rendering them unremarkable and difficult to contest [43,44]. A parallel can be found in Zietz and Das’s qualitative study of young men in Mumbai, India, who collectively reframed street harassment as harmless ‘fun’ and relied on group validation to neutralise personal misgivings [9]. In both contexts, the interpretive authority to name SH is not located in formal policy but in the micro-politics of the peer group; when that group normalises boundary-crossing, individual discomfort is silenced.

### Navigating power relations and organisational management

The image of the university that emerged from our data is one in which formal and informal power has direct and intersecting effects on SH. Formal power structures included the siloed nature of departments and the power invested in individuals. These were further exacerbated by the promotion criteria that looked more at achievement then leadership skills. Informal power was found among those with large research grants or long academic careers, or those who had previously held positions at the university and who had strong networks.

In this context, employees at the university expressed difficulties in navigating behaviours that felt inappropriate but were not clearly sanctionable. These sorts of acts occupy what Taylor [45] calls the ‘yellow zone’, acts that generate discomfort yet remain plausibly deniable and, crucially, rarely trigger formal sanction. The structural logic underpinning this silence is recognised in the harassment literature, with a survey study conducted among university faculty, staff, and students by O’Hare and O’Donohue arguing that organisations often overlook misconduct when perpetrators deliver valued outcomes [46]. The persistence of these ‘yellow zone’ behaviours thus signals not a deficit of rules but a misalignment of incentives, with the same performance metrics that bolster institutional prestige simultaneously deterring colleagues from challenging borderline conduct. Moreover, these seemingly trivial behaviours can nonetheless contribute to significant psychological ill-health and lay the groundwork for more severe harassment. This is illustrated in the meta-analysis by Sojo et al. (2015), that demonstrated how frequent low-intensity gender harassment is associated with increased anxiety, depression, and reduced job satisfaction [47]. Cortina and Areguin further argue that such micro-level slights normalise hostility towards marginalised genders, creating permissive climates that facilitate escalation into overt sexual harassment and broader gender-based violence, thereby underscoring the imperative to counteract these behaviours at their inception [48].

One key consideration concerns manager’s own perception of their remit. Many report feeling powerless to act unless a formal complaint has been filed, yet this belief is not strictly accurate. Under current legislation, organisations are explicitly required to work proactively to prevent harm, rather than merely react once an incident has been reported [20]. In practice, however, managers feel constrained to “react” and insist on a report from the affected individual before taking any steps. This represents a clear mismatch between their mandate to engage in proactive safeguarding, and their experienced responsibility, which they perceive as purely reactive.

Participants’ reluctance to escalate such incidents reflects how they weighed up procedural opacity, career dependency, and reputational stakes. In an interview study conducted among HR managers in Sweden, Cicerali found that the majority of victims they had met preferred quiet mediation over formal investigation [14]. Informal power can also be reflected in gender norms. Gender disparities and imbalances in workforce composition were discussed as a contributing factor for SH in this study, and this aligns with observations from Jordanian campuses that found that distal gender norms that were prevalent in society were able to hollow out ostensibly robust policies through protecting perpetrators, preventing reporting, victim blaming, and discouraging bystander intervention [8].

### Facing a culture of silence and normalisation

The findings of this study depict silence not as resignation but as a deliberate risk-management strategy where employees weigh the cost of speaking up against the institutional payoff and often conclude that silence protects careers and collegial ties. In a number of cases participants also expressed how they would consider whether the harassment had been serious enough. In a qualitative study of discursive interpretation of organisational policies around SH, these sorts of binary logics were found to undermine policy implementation and failed to match the lived experiences of employees [13]. Such strategic silence can also be understood through the idea of ‘non-performative’ institutions, i.e., organisations, that claim to care but implement policies that do not address power imbalances [49].

Silence is sustained by a repertoire of rationalisations that circulate through both policy language and everyday exchange. Employees explain boundary-testing behaviour as generational (“he’s old-school”), cultural (“that’s just how it’s done in our field”) or intent-based (“he meant it as a compliment”), echoing Hardt et al.’s [36] cross-national evidence that such justifications blunt the urge to act.

A substantial empirical literature positions organisational climate as the strongest antecedent of SH; Fitzgerald, Drasgow et al.’s [5] integrated model and subsequent meta-analysis [50] demonstrate that perceived tolerance predicts both prevalence and harm. The present study advances that work by illuminating the mechanics through which this climate is reproduced in the university setting. This includes the ways in which performance metrics at the university act as incentives and thus shield high-performing perpetrators from consequences, opaque procedures and hierarchical “gatekeepers” that make reporting a potential risky business, and untrained leaders that default to inaction, together sustaining a ‘yellow zone’ where boundary-pushing conduct is rarely sanctioned.

Organisational research indicates that cultures of silence can be disrupted when harassment prevention is woven into bystander-focused training and linked to clear reward or sanction systems. Experimental and field studies show that giving employees concrete intervention scripts and publicly recognising those who act increases the likelihood that peers challenge boundary-testing behaviour [51,52]. Longitudinal panel evidence collected on corporate affirmative action policies further suggests that norm change is most durable when zero-tolerance expectations are embedded in appraisal and promotion criteria, signalling that leadership will back words with material consequences [53].

### Offering peer support for collective action

Participants in the study stressed that informal peer networks often provide the first, and sometimes only, line of defence for individuals who feel harassed. These “back-channel” practices mirror the peer-to-peer safety nets Löfgren et al. document among Swedish students [10], and the mentor shields that Jenner et al. observed in hospital teams [54]. Such micro-acts demonstrate a sense of collective responsibility that operates outside prescribed procedures and can, at least temporarily, reduce victims’ exposure. In the absence of formal systems that are trusted, these informal channels, together with contextual factors in the local environment, can be an important element of prevention and response. These results are echoed by those of Handy’s study of ‘communities of coping’, showing that among women in a small New Zealand town, where informal support, alongside organisational culture and the local environment, was found to be a strategy developed to combat workplace SH [55].

Yet bystander theory suggests that willingness to intervene is heavily contingent on perceived personal risk and situational clarity [51]. Our data confirm those contingencies where employees expressed how they would be more hesitant to respond if the perpetrator outranked them, when norms were ambiguous, or when previous helpers had faced backlash. This aligns with large-scale campus studies showing that fears of social sanction sharply curtail bystander action even when moral motivation is high [52,56].

### Strengths and limitations

This study used focus group discussions for data collection, a method well suited to studying norms and attitudes as well as supporting research into sensitive topics [57]. Focus group discussions also allowed the researcher to observe the dialogic process of understanding and navigating workplace norms and organisational culture. This allowed the research team to identify excerpts of conversations to show how the categories and sub-themes are grounded in the data, thus enhancing the credibility of the results. The use of a robust analytical procedure according to the methods described by Graneheim and Lundman [22] and further expanded by Lindgren [58] and keeping a clear documentation of the research process through the use of notebooks and seating plans improved dependability. The emphasis on achieving consensus between all authors of the article contributed towards confirmability. Finally, by providing clear descriptions of the study environment and context the transferability of the results was improved.

In addition, the inclusion of both employees with management/leadership responsibilities and those without (in separate discussion groups) enhanced the authenticity of the findings ensuring that complexity and variation in the points of view were included. This in turn further contributed to the transferability of the results.

The authors of this article are connected to Lund University through employment or studies. This gives them a unique perspective as ‘insiders’. Conducting research as an insider offers both advantages and challenges. On the one hand, insider status can provide contextual understanding and facilitate access [59], but it also raises concerns about role conflict, confidentiality, and potential bias [60]. Reflexivity and inter-author dialogue were essential in navigating power dynamics and ensuring methodological rigour.

Limitations to this study include the sampling procedures that may have led to those most interested in the topic attending, thus missing out on other important perspectives. Bearing in mind the fear of consequences of reporting SH raised in this study, certain employees at the university could have chosen to avoid participating due to the nature of the topic. In addition, some participants (n = 4) did not attend the focus group discussions (FGDs) for which they had registered, primarily due to scheduling difficulties. As a result, several FGDs were conducted with fewer than optimal numbers of participants, and in one case only two participants were present. Conducting FGDs with smaller group sizes may have restricted the range of experiences and perspectives captured within those discussions and may have limited opportunities for variability and contrasting interpretations to emerge through group interaction. Findings from these smaller groups should therefore be interpreted with appropriate caution, particularly with regard to breadth and transferability.

At the same time, methodological accounts in the qualitative literature acknowledge that smaller FGDs can still generate meaningful interaction and data where participants engage actively with one another for example [61]. In our study, the FGD involving two participants generated sustained discussion and mutual interaction rather than parallel individual accounts. Its content was not analysed in isolation but interpreted in relation to patterns observed across multiple FGDs conducted with different group compositions. Excluding these data was therefore considered to risk introducing other analytic and ethical limitations.

The authors also recognise the complex and varied nature of different workplaces at Lund University. To protect the identity of the participants this information was not collected. This could, however, create uncertainty about whose voices are being heard, and how these discussions should be interpreted.

Focus groups can also suffer from social desirability bias and group biases that limit dissenting voices, especially around sensitive topics [62]. Indirect questioning and abstraction of framing, as is commonly found in focus group discussions, however, have been shown to minimise these biases [63].

### Implications for practice and further research

This study reveals an articulated need among university employees to develop shared understandings and a common language when it comes to sexual harassment. Employees, both in managerial positions and those without, struggle when talking about a phenomenon where perceptions are influenced by gender, culture, legal definitions and personal experience. To contribute to a shared understanding of what can be tolerated and not, a university wide code of conduct, focusing not specifically on sexual harassment, but more broadly on respectful behaviour and highlighting zero-tolerance of any uncivil or harassing behaviour, might be useful. Sustained dialogue is also needed to translate abstract principles into local practice. Regular discussions and joint reflection sessions could help to make visible hidden norms and help align employees around a common threshold of acceptable behaviour. Without this iterative work, even the best-written code of conduct or policy, risks remaining a document rather than a living standard.

Swedish employers have a *proactive* legal duty under both the Discrimination Act [17] and the Work Environment Act [19] to prevent harassment and investigate any signs of it. This study adds to a growing body of evidence demonstrating how workplace climate and collective norms can either enable or restrain harassing behaviours [50]. Our findings suggest that universities should move beyond interventions aimed solely at individual victims or perpetrators, but instead prioritise preventative strategies that shape the overall work-environment climate. In practice, this means embedding collective responsibility for fostering a culture in which harassment is neither tolerated nor ignored and equipping all staff with bystander skills so they can recognise and intervene in inappropriate behaviour as it occurs [64].

At the same time, institutions should streamline and demystify formal reporting channels, which participants in our study often perceived as opaque and driven more by reputational concerns than by a genuine commitment to justice. Crucially, organisations cannot wait for formal complaints to drive change, since many employees struggle to recognise and label harassing behaviours. Thus, prevention must focus on improving the social climate itself. Indeed, evidence suggests that enhancing overall workplace climate is among the most effective means of reducing the incidence of sexual harassment [65]. Not only does this align with best practice, but it also fulfils Swedish legal requirements for systematic work-environment management.

Another key finding from the results was that employees in managerial positions felt that they lacked the necessary training, while also being torn between collegial loyalty and legal responsibility, amplifying the so-called ‘yellow zone’ vacuum where ambiguous behaviour persists. This feeling was shared by employees not in managerial positions who saw a vacuum in leadership skills. Targeted training should combine emotion-focused skills such as how to respectfully receive disclosures without defensiveness, with clear guidelines for how any investigation should occur. This could also be achieved through external ‘experts’ who could be brought in to support managers in this task.

The results of this study contribute to a deeper understanding of the perceptions of culture and norms surrounding sexual harassment among employees in an academic workplace in Sweden, contexts in which SH occurs, and in which these behaviours are, or are not, reported. The results also highlight a need for further research into how best to create a workplace culture where these behaviours are not accepted as well as how to challenge the culture of silence. This could be, for example, an evaluation of interventions to improve bystander support, longitudinal studies to examine the impact of providing additional support to managers on workplace norms and reporting, and studies that explore the impact of interventions specifically targeted at workplace climate.

## Conclusion

Sexual harassment in academia is not simply an issue of policy or awareness, but one of culture, climate, silence, and negotiated responsibility. Our study contributes to a growing body of work showing that SH is maintained not only through overt acts, but through everyday ambiguity, formal and informal hierarchies, and a variety of organisational responses. Addressing these challenges requires more than symbolic policy compliance. It calls for proactive measures contributing to a shared understanding of unacceptable behaviours based on continuous training and development in order to create a work environment in which these behaviours are detected and prevented.

## Supporting information

S1 File Thematic FGD guide navigating the unspoken. (DOCX)
