# Supplementary material for: Navigating the unspoken: A qualitative study of employees’ perceptions of culture and norms surrounding sexual harassment at a Swedish university
Source: PLoS One. 2026 Jun 16;21(6):e0351724. doi: 10.1371/journal.pone.0351724 (PMC13271518; doi:10.1371/journal.pone.0351724)
Supplement: S1 File — (DOCX) [file pone.0351724.s001.docx]

**Occurrence of sexual harassment**

Form or expression

Perceived causes

Consequences

**Expectations for the future**

Suggestions for future prevention/response

Responsibility for implementation

**Work environment**

Discourse in the workplace

Norms around harassment

Existing forms of support

**Organisational culture**

Dependency

Power relations

Opportunities for reporting

**What is sexual harassment**

Norms and attitudes (MeToo)

Definition

Expression

Thematic guide for Focus Group Discussions among employees at a large university in Sweden.
