# Supplementary material for: Navigating the unspoken: A qualitative study of employees’ perceptions of culture and norms surrounding sexual harassment at a Swedish university
Source: PLoS One. 2026 Jun 16;21(6):e0351724. doi: 10.1371/journal.pone.0351724 (PMC13271518; doi:10.1371/journal.pone.0351724)
Supplement: Attachment — Submitted filename: Response to Reviewers Navigating the unspoken Final.docx [file pone.0351724.s004.docx]

**Authors’ response to editor and reviewer’s comments**

**Title:** Navigating the unspoken: a qualitative study of employees’ perceptions of culture and norms surrounding sexual harassment at a Swedish university

**Author names:** Jack Palmieri, Frida Pilgaard, Anette Agardh

Version: 2

**Date:** 2026-03-24

Authors’ response to reviewers: See the following pages.

Dear Kshitij Karki,

We would like to thank you and the reviewer(s) for the valuable feedback. We have revised the manuscript in line with this feedback. Please see the revised version attached and our responses to the reviewers’ comments, point by point, below.

With kind regards,

Jack Palmieri

**Comments from Reviewer #1**

1. Thank you, PLOS ONE for the opportunity to review this manuscript reporting employees’ perceptions of culture and norms surrounding sexual harassment at a Swedish university. Please find my comments and recommendations below.

**Response:** We thank the reviewer for their time and efforts.

2. Kindly suggesting reducing the word count, the manuscript is a bit lengthy and there is room for concise communication throughout the manuscript to enhance readability and brevity.

**Response:** We thank the reviewer for this comment. We have shortened the manuscript in a number of areas, most significantly in the introduction section.

3. The introduction can be strengthened by maintaining a flow of ideas that are linked from one paragraph to the next, clearly articulating the problem being researched. Currently the introduction seems quite long with ideas that are not flowing so well together. Suggesting framing the introduction in this manner. Firstly, providing background context-clearly describing the situation that gives rise to the problem. Secondly, clearly stating the problem that exist. Thirdly, explaining why the problem matters-the consequences if the problem is not addressed. Fourthly, indicating the knowledge gap and how your research will help fill the gap or solve the problem.

**Response:** We thank the reviewer for this comment. We have streamlined the introduction both for brevity and flow of ideas as you suggest.

4. Line 24: Define “recent”-if possible, provide the dates of the meta-analyses for better understanding of when the meta-analyses were done.

**Response:** We thank the reviewer for this comment. The meta-analyses in question were by Latcheva from 2017 and Debenath 2025. This information has now been added to the manuscript.

Lines 42 and 43

*With recent meta-analyses by Latcheva (2017) and Debnath (2025) confirming that it remains a serious and under-addressed form of gender-based violence [2, 3]*

5. Line 75: Please provide reference for this statement.

**Response:** We thank the reviewer for this comment. The correct reference has now been added.

6. Line 75: Kindly elaborate the multiple countries, are they from the same continent or different continents? European, Asian etc

**Response:** We thank the reviewer for this clarification. The cited reference was conducted in Belgium, Croatia, Germany, and Sweden. This has been clarified in the manuscript in the following way

*In European settings, uncertainty regarding definitions, reporting channels, and access to support has been shown to impact reporting and prevention*

7. Line 90: Define “recent”-if possible, provide the dates of the systematic review for better understanding of when the review was done.

**Response:** Thank you for this suggestion. The systematic review dates from 2024. This has now been added to the manuscript as follows

*A 2024 systematic review of gender-based violence in academia found that over half of university staff had experienced some form of interpersonal violence, with women and LGBTQ+ staff reporting disproportionate exposure to SH, stalled career advancement, and reduced psychological well-being [14]*

8. Line 105: Put an apostrophe symbol on employees (employees’)

**Response:** We thank the reviewer for this; the apostrophe has been added.

9. Line 112: Remove current

**Response:** We thank the reviewer for this, current has been removed, and the sentence reformatted as follows:

*In order to fill these knowledge gaps, this study aimed to explore how employees at a Swedish university perceive the organisational culture and workplace norms surrounding sexual harassment.*

10. Line 112-113: Indicate that this is a qualitative study.

**Response:** We thank the reviewer for this, the term qualitative has been added as follows:

*To address these gaps, this qualitative study aimed to explore how employees at a Swedish university perceive the organisational culture and workplace norms surrounding sexual harassment.*

**Methods**

11. Line 179-196: Kindly move Ethical Considerations to be the first paragraph in the methods section.

**Response:** We thank the reviewer for this suggestion. As several elements discussed in ethical considerations relate to information first introduced in the methods section, however, we have chosen to keep the ethical considerations section where it is in the manuscript to enhance flow and improve readability.

12. Please add a subheading study population, location and sampling before data collection.
    a. Add the following points under this topic
    -Defining eligibility criteria for participation in the FGDs
    -Study locations where FGDs were held
    -Participants sampling (was it purposive, random etc)
    -Description of the each FGD participants (how many in each group, the gender composition, was it mixed departments in each FGD)

**Response:** We thank the reviewer for the comment. We have now added a section named study population and sampling. In this section we have described the study setting, clarified the eligibility criteria and sampling. Information regarding how the focus groups were comprised has been clarified but kept under data collection.

13. Line 124-141: This belongs in the introduction section as it is providing background information which is helpful to the international audience.

**Response:** We thank the reviewer for this suggestion. We agree that information regarding the university and its students and staff are important for any audience. However, we see study setting as being the most relevant section for this as it pertains to the specifics of this study rather than the broad background of the topic. However, the section has been shortened and moved to study setting and, in line with your comment 14, key figures have been added to the introduction.

14. Line 140: Kindly elaborate on the key findings: what was the prevalence of SH, what were the characteristics of SH, does these key findings provide justification of the research problem.

**Response:** We thank the reviewer for this suggestion. These key findings have been elaborated upon in the introduction in line with comment 13.

*In the Swedish academic context, a 2019 survey of staff at conducted as part of the Tellus project at Lund University showed that 25% of female employees and 7% of male employees report experiences of SH during their employment [19]. When limiting the response period to the past 12 months, the corresponding figures were around 8% of female employees, and 3% of male employees [19].*

15. Line 142: The use of the word current study is sort of confusing, does this imply that the study is still ongoing?

**Response:** We thank the reviewer for this comment. The word current has been removed from this sentence.

16. Please clarify the criteria for participation in the study so that it becomes clear. As it reads now, what does all employees mean. Is it all university employees, is it employees from a specific department? This falls under study population, location, sampling subheading.

**Response:** We thank the reviewer for this comment. Participation was open to all employees of the university regardless of department, position, type of employment or experience of SH. The information has been added to the new section as suggested.

17. Line 142-143: Belongs in the study population, location, sampling subheading. Please elaborate more on the recruitment, where the posters e-posters, where were they placed at the university? How many people expressed interest to participate in the FGDs? Did everyone who expressed interest end up participating in the FGDs, if not how was sampling done for the participants who participated.

**Response:** We thank the reviewer for this comment. Posters were physical and displayed in all faculties of the university. Internal communication channels including email was used to distribute the information to all employees using university email lists. All respondents who expressed interest in participating were offered a place in an FGD. Not all participants who signed up for an FGD attended. This information has been added to the manuscript

18. Line 148: Kindly provide reference for the guide, is it in Appendix?

**Response:** We thank the reviewer for this suggestion. The FGD guide has been added as an appendix.

19. Line 157: Kindly provide clarity on the justification for having 10 FGDs with some FGDs having 2, 3 participants. Were all these FGDs conducted at Lund University, was it not possible to have less FGD with a reasonable number of participants in each FGD. How did you select the participant in each FGD?

**Response:** We thank the reviewer for this comment. Following the recommendations in guiding literature, and our own experiences of booking and conducting FGDs, our aim was to conduct FGDs with 5-8 participants in each. Practical considerations, however, regarding booking of participants, finding time slots that worked, and ensuring those with managerial responsibility were separated from those without, led to some FGDs being booked with 4-6 participants. In a number of cases, participants did not turn up (n=4), and the FGDs were therefore conducted with the numbers shown in the manuscript. This was of course regrettable but considered preferable to cancelling the FGD at the last minute. This issue has now been clarified in the strengths and limitations in the Discussion.

*In addition, some participants (n=4) did not attend the FGDs they had booked. These participants primarily gave scheduling challenges as the reason, but the result was that several FGDs were conducted with less than optimal numbers of participants.*

20. Line 166: Table 1, participant characteristics are usually presented under the Results sections as the first point describing the key demographics of the participants. Kindly provide the age ranges of the participants. Other than the sex and language did you collect any other demographics information for the participants?

**Response:** We thank the reviewer for this suggestion. Participant characteristics have been moved to the results section. No demographic data were collected on the participants beyond what is already presented here. This was a deliberate strategy to further protect the identities of the participants considering the potentially sensitive nature of the questions.

21. Line 166: Table 1, FGD 8 only had 2 participants. Ideally an FGD leverages on the group dynamics and interpersonal interactions among multiple people in-order to generate broad range of ideas. I am not sure if this was achieved by having only 2 participants, could you provide justification for conducting an FGD with only 2 people.

**Response:** We thank the reviewer for this and agree that FGDs must always leverage the interaction between participants. As explained in answer 19, no FGDs were planned with 2 (or even 3) participants, but the decision was made to continue with these even when participants did not attend. The facilitator is experienced in conducting FGDs and felt that the information collected during this FGD was equally valuable to include in the analysis.

22. Line 168, who transcribed the audio recordings? The audio recording in Swedish where they translated to English? Kindly elaborate.

**Response:** The audio recordings were transcribed verbatim in Swedish by a research assistant. Translation to English took place during the coding process by the first author. This has also been clarified in the text as below.

*All FGDs were audio recorded and transcribed verbatim by a research assistant at the university. The analysis followed the principles of qualitative content analysis according to Graneheim and Lundman [20]. Firstly, all transcripts were read through multiple times to gain familiarity with the data. Following this, meaning units relevant to the research aim were identified and coded inductively in English by the first author. These codes were then grouped into sub-categories and categories through a combination of inductive and deductive processes, moving iteratively between the empirical data and emerging interpretations.*

*Categories were subsequently examined for the development of latent sub-themes through a process of pattern recognition and conceptual reflection. Finally, one overarching theme was formulated. Quotes were selected to illustrate the categories and these were translated to English by the first author. The final model was developed collaboratively, with all co-authors reviewing coding decisions and contributing to analytical refinement.*

23. Line 195: Were the audio-records deleted from the audio-recorders?

**Response:** All recordings were deleted from the recorders immediately after they had been transferred to the encrypted hard drives which were kept locked in a secure safe at the university. This has been clarified in the manuscript.

*All identifying information was removed during the transcription process to protect participant anonymity. All audio recordings were transferred to encrypted hard drives immediately after the FGD and deleted from the audio recorders. These audio recordings and transcripts were securely stored on encrypted drives with access restricted to members of the research team. Consent forms were stored separately in a locked safe.*

24. Line 181: You have already spelt out the acronym in line 145.

**Response:** Thank you for noting this – this has now been rectified in the manuscript.

**Results**

25. Line 200: Suggesting presenting the sentence in this manner; ‘…that presents perceptions of Swedish university employees on the organisational culture and workplace norms surrounding SH.’ Themes are showing us what they think about SH.

**Response:** We thank the reviewer for this suggestion. We have re-written this sentence as follows.

*The analysis of the focus group discussions resulted in one overarching theme, four sub-themes, and eight categories that reflect how Swedish university employees perceive the organisational norms and workplace culture surrounding*

26. The results section is a bit difficult to follow, the presented themes are a bit broad and more descriptive rather than analytical.

**Response:** We thank the reviewer for this feedback. However, we feel that the presented results are an accurate reflection of the analysis process and represent the correct level of abstraction and interpretation.

27. Line 216: Is there supposed to be a Figure 1 inserted here?

**Response:** We thank the reviewer for this comment. Following the submission guidelines for PLOS One we have not put any of the figures in the manuscript file but rather submitted them as separate images. Hence the placeholders and heading are left, but the figures themselves are in a separate file.

28. Line 218: Kindly rephrase, following section implies you are moving from the results section. Maybe say following paragraphs.

**Response:** We thank the reviewer for this comment. We have changed this in the l

Text to “following paragraphs”

29. There is mention of the #MeToo a couple of times in the results and discussion section, however in the introduction you have not provided much background on what it is about.

**Response:** We thank the reviewer for this feedback. We feel that the #MeToo campaign was sufficiently global to not require significant description. To support readers who may not be acquainted with the movement, however, a reference has been added in the introduction as follows.

*Denmark found that employees often struggled to label their experiences as SH, a process shaped by peer responses, cultural discourse, and moments of public reckoning such as the #MeToo social movement [10,11].*

30. Line 364: What is an Ostrich effect?

**Response:** The ostrich effect is the effect of deliberately avoiding, ignoring, or rejecting negative information to protect themselves from psychological discomfort through ‘burying one’s head in the sand’. A short text has been added to explain this.

*In some cases, negative experiences with leadership were discussed, with heads of department exhibiting an ‘Ostrich effect’ whereby they ‘bury their heads in the sand’ and ignore any information or issues that might cause them discomfort (FGD 7, M).*

31. Line 376: What is ‘Scandinavian complacency’? Its good practice to explain phrases in a manuscript as some readers may not be familiar with them.

**Response:** Scandinavian complacency refers to the complacency that comes from a security in the stability of societal services including welfare and the perception of flat hierarchies that suggest power dynamics are not an issue in this part of the world. This has now been clarified in the text

*These hierarchies could be related to who has research funding, or who has been employed the longest, and were seen as being hidden by the idea of flat hierarchies and the perceived security due to societal stability referred to by one of the participants as ‘Scandinavian complacency’ (FGD 7, M).*

32. Line 443: Put a full-stop after the sentence.

**Response:** Thank you, this had been added.

**Discussion**

33. Line 635: Provide the dates of the meta-analysis and the author.

**Response:** The name and date of the meta-analysis has been added as follows

*A meta-analysis of U.S. studies conducted in 2003 by Ilies et al. reveals substantial variation in reported harassment rates across industries and demographic groups, suggesting that national norms, workplace cultures and individual backgrounds influence whether similar behaviours are labelled as harassment [31]*

34. In the discussion section, suggesting you focus on the key issues coming out of the results section and mapping the way forward rather than discussing all issues presented in the results sections.

**Response:** We thank the reviewer for this suggestion. While we have structured the discussion according to the sub-themes from the results, only key topics are presented.

35. Line 767: This section is the study strengths and limitations, present it as such rather than methodological considerations.

**Response:** We thank the reviewer for this comment. We agree that, with its focus on trustworthiness, it is more akin to a strengths and limitations, we have therefore renamed it as such.

36. Line 789: You start by saying other limitations, but you have not mentioned any limitations for the study.

**Response:** We thank the reviewer for this comment. The section has now been amended.

**Comments from Reviewer #2**

1. It is important to take into account both content and thematic analysis. So Granheim alone does not suffice.

**Response**: We thank the reviewer for this comment. We agree that both thematic analysis and qualitative content analysis are established approaches for analysing qualitative data. In the present study, however, we deliberately chose qualitative content analysis according to Graneheim and Lundman, as it provides a systematic and rigorous framework for analysing both manifest and latent content, while allowing for identification of similarities and variations across participants. Given the study aim and the analytic procedures applied, we consider Graneheim and Lundman’s method of qualitative content analysis sufficient to address the research question. We therefore did not see a methodological or practical advantage in combining analytic approaches.

2. Clarify the qualitative study more- can it fit as an interpretive phenomenological analysis?

**Response:** We thank the reviewer for this suggestion. While interpretive phenomenological analysis is well suited for in-depth exploration of individual lived experiences, it is not methodologically aligned with the present study and its focus on the group level. Our aim was to explore shared perceptions of organisational culture and workplace norms surrounding sexual harassment, based on focus group discussions that emphasise interaction and collective meaning-making.

The study therefore applies qualitative content analysis which is appropriate for analysing both manifest and latent content across participants and for examining socially constructed organisational phenomena.

3. Study data cannot be shared ethically , but interview transcripts can be uploaded in the repository

**Response:** We thank the reviewer for this suggestion. The focus group discussion transcripts constitute the primary data of this study and cannot be made publicly available. The data were collected from a small, potentially identifiable group and include sensitive information. Participants did not consent to public sharing of de-identified transcripts beyond the research team, and such sharing would conflict with the terms of the ethical approval.

In accordance with Swedish legal requirements and the conditions set by the Swedish Ethical Review Authority, access to the underlying data is therefore restricted. Reasonable data requests may be directed to Lund University via [*registrator@lu.se*](mailto:registrator@lu.se) and will be subject to ethical and legal review. This has been clarified in the submission system as follows:

*The interview transcripts and associated personal data cannot be made publicly available because they were collected from a small, potentially identifiable group and contain sensitive information. Participants did not consent to sharing their de-identified data beyond the research team, and doing so would conflict with the terms of our ethical approval. Access to the underlying data is therefore restricted in accordance with Swedish legal requirements and the conditions set by the Swedish Ethical Review Authority. The data are securely deposited at Lund University (Sweden). Data requests may be made to Lund University via registrator@lu.se and will be subject to review and approval by the Ethical Review Authority.*

4. IRB letter can also be uploaded- from Malmo and Lund university

**Response:** We thank the reviewer for this comment. In the Swedish research ethics system, universities do not operate local Institutional Review Boards (IRBs). Instead, ethical approval is granted by a national authority. This study was reviewed and approved by the Swedish Ethical Review Authority (reference number: 2018/350).The ethical approval decision and associated documentation are publicly available.

5. How was the data analyzed?

**Response:** We thank the reviewer for the opportunity to clarify the analytic procedure. The data were analysed using qualitative content analysis following the approach described by Graneheim, Lundman and Lindgren (2004, 2020). All transcripts were read repeatedly to gain an overall understanding of the data. Meaning units were then identified and coded. Codes were grouped into sub-categories and categories based on similarities and differences. Finally, an overarching theme was formulated on a latent level. This analytic process is described in greater detail in the Methods section (lines 168–177).

6. A different method, such as a Delphi group for example, would be more appropriate than IPA to seek agreement among experts

**Response:** We thank the reviewer for this suggestion. We agree that a Delphi study is well suited for achieving consensus among experts. The aim of the present study, however, was to explore shared perceptions of organisational culture and workplace norms surrounding sexual harassment among employees, rather than to seek expert agreement or consensus.

The study therefore used focus group discussions, which emphasise interaction, dialogue, and collective meaning-making, elements that are central to understanding organisational culture. Participants were not recruited as experts, and consensus was not an analytic goal. For these reasons, a Delphi approach was not considered methodologically appropriate for this study.

7. It is important to give more demographic profiling

**Response:** We thank the reviewer for this feedback. We agree that information about research participants is important for contextualising and interpreting qualitative findings. However, given the sensitivity of the topic and the organisational setting, we deliberately limited the collection of demographic data to protect participant anonymity. Recruitment was open to all employees at the university, regardless of employment type, age, or country of origin. The only demographic information collected concerned managerial responsibility, which was necessary to ensure that participants were not placed in focus groups with their direct superiors. Information on language refers to the language used in the focus group discussions, not to participants’ backgrounds. We also chose not to compare faculties or units.

While additional demographic data might have allowed for further subgroup analyses, this was not compatible with the ethical considerations and study aims.

8. please follow APA guide as to how to write experiential and exponential statements when reporting interviewed statements

**Response:** We thank the reviewer for this comment. By clearly marking the quotes (and including verbatim quotes) in the manuscript we adhere to this separation. In the analysis, as discussed in the methods section, themes are on a latent level (researcher interpretation) while categories and subcategories remain on the manifest level.

9. references need to be updated,

**Response:** We thank the reviewer for this feedback. We have reviewed the reference list and made a number of minor updates to ensure they are correct.

10. out of context references are mentioned, e.g 13- harassments in Indian context.

**Response:** We thank the reviewer for their diligence. The reference was included as an illustrative example from the limited literature on peer affirmation and collective normalisation of harassment, rather than as a direct contextual comparison. The study context was already specified as Mumbai in the Discussion (line 680); following the reviewer’s suggestion, we have now explicitly added India to ensure clarity.

The revised sentence now reads

*A parallel can be found in Zietz and Das’s qualitative study of young men in Mumbai, India, who collectively reframed street harassment as harmless ‘fun’ and relied on group validation to neutralise personal misgivings [13].*

**Comments from Reviewer #3**

1. I have thoroughly reviewed your manuscript and commend its valuable contribution to understanding sexual harassment among employees in tertiary institutions. It is well written, with a background that effectively situates the issue within both global and local contexts. The problem is clearly defined, and the research questions are well articulated and aligned with the central issue under investigation.

   The study methodology is clearly articulated, with an appropriate design, sampling strategy, and data collection methods that align well with the research problem under investigation. The discussion is well developed, highlighting the significance of the study and contextualising the findings while drawing meaningful comparisons with previous research both globally and locally.

   The strengths and limitations of the study are clearly articulated and the ways in which these were addressed are well explained. The implications of the findings for managing sexual harassment among employees in tertiary institutions are thoughtfully discussed, along with considerations for future policies and interventions. However, here are a few minor comments on aspects that deserve your attention to further improve the article.

**Response:** We thank the reviewer for their kind feedback on this manuscript.

Methods

2. Lines 124-141: Please separate sample/sampling procedures from data collection procedures by creating separate subheadings under methods section. This makes it easier for readers to follow.

**Response:** Thank you for this suggestion, we have now revised as suggested and have a subheading for Study population and sampling and one for Data collection.

3. Lines 153-154: Suggesting that you briefly describe Malmö University, where it is located, student enrolment and staff; this helps readers understand how the pilot university resembles the university under study.

**Response:** We thank the reviewer for this suggestion. Malmö University is located in Southern Sweden, very close to Lund University. The university has five faculties and enrols approximately 28,000 students. The university staff number approximately 2,00. We have added a short description (line 155) to the manuscript to this end. The new next reads:

*Located in southern Sweden, close to Lund University, Malmö University employs around 2,200 staff and enrols 28,000 students across five faculties. International employees make up about 20% of staff [26].*

Results

4. Include data collection timelines: when was data collected?

**Response:** Thank you for this response. Data was collected as part of the Tellus project during the period February to May 2019. This has now been added to the manuscript line 144 as follows.

*Recruitment was carried out from November 14^th^, 2018, to May 7^th^, 2019, and data collection from February to May, 2019*

5. Did you have international staff/employees participating in the group discussions? How many? If yes you could include this under participant characteristics.

**Response:** We thank the reviewer for this suggestion. We agree that information about research participants is important for contextualising and interpreting qualitative findings. However, given the sensitivity of the topic and the organisational setting, we deliberately limited the collection of demographic data to protect participant anonymity. Unfortunately, therefore we do not have information on whether the participants were international employees or not. Even information on language refers to the language used in the focus group discussions, not to participants’ backgrounds.
