# Supplementary material for: Navigating the unspoken: A qualitative study of employees’ perceptions of culture and norms surrounding sexual harassment at a Swedish university
Source: PLoS One. 2026 Jun 16;21(6):e0351724. doi: 10.1371/journal.pone.0351724 (PMC13271518; doi:10.1371/journal.pone.0351724)
Supplement: Attachment — Submitted filename: Reviewer 3 comments PONE-D-25-35396R1.docx [file pone.0351724.s003.docx]

**Comments: Reviewer #3**

Thank you for sharing this revised manuscript for further review and comments. I would like to appreciate the authors’ efforts in revising this manuscript in line with reviewer comments. While authors have adequately addressed most comments raised by reviewers, I bring their attention to a few comments below:

1. Check punctuation through the whole manuscript, for example:
   - Line 51**:** Place a comma after to: […] to organisational climate.
   - Line 54-56: Place a comma after SH […] not only individual incidents of SH but also the wider organisational norms […]
   - Line 57-58: Place a comma after challenged: […]. whether SH is acknowledged, challenged, or normalised.
2. Discussion:

- Address capitalisation for consistency across headings through the whole manuscript e.g., line 563: Facing a culture of silence and normalisation or line 707: Negotiating Collective Norms and Meanings.
- Line 714: Suggesting you state who coined the Classic Symbolic Interactionist Theory and year.
- Line 896: It is suggested that the authors elaborate further on how conducting FGDs with fewer than optimal numbers of participants may have affected the quality of the study, for example a narrower range of experiences may have been captured, limitations in the transferability of findings, and other impacts extending beyond participant interaction alone. This is a major limitation of this study.
