# Supplementary material for: Navigating the unspoken: A qualitative study of employees’ perceptions of culture and norms surrounding sexual harassment at a Swedish university
Source: PLoS One. 2026 Jun 16;21(6):e0351724. doi: 10.1371/journal.pone.0351724 (PMC13271518; doi:10.1371/journal.pone.0351724)
Supplement: Attachment — Submitted filename: Response to Reviewers Navigating the unspoken_Review 2.docx [file pone.0351724.s005.docx]

**Authors’ response to editor and reviewer’s comments**

**Title:** Navigating the unspoken: a qualitative study of employees’ perceptions of culture and norms surrounding sexual harassment at a Swedish university

**Author names:** Jack Palmieri, Frida Pilgaard, Anette Agardh

Version: 3

**Date:** 2026-05-06

Authors’ response to reviewers: See the following pages.

Dear Kshitij Karki,

We would like to thank you and the reviewers for the valuable feedback once again. We have revised the manuscript in line with this feedback. Please see the revised version attached and our responses to the reviewers’ comments, point by point, below.

With kind regards,

Jack Palmieri

**Comments from Reviewer #1**

1. No Further comments

**Comments from Reviewer #2**

1. Most of the comments were met, but the Delphi approach would have improved the study.

**Response:** We thank the reviewer for this suggestion. We agree that the Delphi approach is a powerful method for achieving expert consensus. Its focus on iteration, control and expert opinion make it an appropriate method to use for certain research questions. The aim of the present study, however, was to explore shared perceptions of organisational culture and workplace norms surrounding sexual harassment among employees, rather than to seek expert agreement or consensus. The study builds on existing research that captures the discursive and collaborative nature of culture and norms.

The study therefore used focus group discussions, which emphasise interaction, dialogue, and collective meaning-making, elements that are central to understanding organisational culture. Participants were not recruited as experts, and consensus was not an analytic goal. For these reasons, a Delphi approach was not considered methodologically appropriate for this study.

2. Please update the literature.

**Response:** We thank the reviewer for this comment. We have reviewed the literature list, updated references where needed (including adding DOIs where possible) and added two additional references, one of which has been published since the reviewing process began. More specifically these references draw on the normalisation of harassment by male students in South Africa, and the related concept of “dangerous common sense” in the same context (Kiguwa et al., 2015; Jagath & Hamlall, 2024). These have been added to the discussion as follows:

*Lines 678-682*

This process of collectively negotiated meaning‑making echoes findings from studies from South African Universities, where focus group‑based research among students and staff has shown how everyday verbal and physical violations are frequently framed as ordinary expressions of masculinity or institutional life (“dangerous common sense”), rendering them unremarkable and difficult to contest [42, 43]

3. Also upload transcripts and related data in repository.

**Response:** We thank the reviewer for this suggestion. In this case The interview transcripts and associated personal data cannot be made publicly available because they were collected from a small, potentially identifiable group and contain sensitive information. Participants did not consent to sharing their de-identified data beyond the research team, and doing so would conflict with the terms of our ethical approval. Access to the underlying data is therefore restricted in accordance with Swedish legal requirements and the conditions set by the Swedish Ethical Review Authority. The data are securely deposited at Lund University (Sweden). Data requests may be made to Lund University via registrator@lu.se and will be subject to review and approval by the Ethical Review Authority.

4. Please explicitly mention which software was used to group data in themes and codes.

**Response:** We thank the reviewer for this comment. Data coding and analysis was conducted using OpenCode 4. This has now been added to the manuscript as follows.

*Lines 161-162*

Data analysis was conducted using OpenCode 4 [26].

**Comments from Reviewer #3**

1. Thank you for sharing this revised manuscript for further review and comments. I would like to appreciate the authors’ efforts in revising this manuscript in line with reviewer comments. While authors have adequately addressed most comments raised by reviewers, I bring their attention to a few comments below:

**Response:** We thank the reviewer for their kind feedback and for their continued efforts to improve the manuscript.

2. Check punctuation through the whole manuscript, for example:
   a. Line 51**:** Place a comma after to: […] to organisational climate.
   b. Line 54-56: Place a comma after SH […] not only individual incidents of SH but also the wider organisational norms […]
   c. Line 57-58: Place a comma after challenged: […]. whether SH is acknowledged, challenged, or normalised.

**Response:** We thank the reviewer for these comments. We have reviewed the punctuation throughout the manuscript and made minor corrections where appropriate. The manuscript follows UK English grammar and punctuation conventions. Having considered the specific examples raised, we believe that the suggested commas are not required under standard UK usage and, in these cases, do not improve clarity. We have therefore not made changes to those instances.

Discussion:

3. Address capitalisation for consistency across headings through the whole manuscript e.g., line 563: Facing a culture of silence and normalisation or line 707: Negotiating Collective Norms and Meanings.

**Response:** We thank the reviewer for this valuable feedback. We have now standardised capitalisation using sentence case to reflect the journal requirements.

*For example:*

Line 164: Ethical considerations

Line 605: Negotiating collective norms and meanings

Line 688: Navigating power relations and organisational management

Line 727: Facing a culture of silence and normalisation

4. Line 714: Suggesting you state who coined the Classic Symbolic Interactionist Theory and year.

**Response:** We thank the reviewer for this response and agree that it strengthens the text. We have added the information as follows:

*Line 613*

Seeing the boundary of what constitutes SH as a negotiated reality mirrors what classic symbolic interactionist theory (coined by Herbert Blumer in 1937 [27] ) terms a negotiated order, an interpersonal process in which actors continuously redefine situations through talk, eye-contact and tacit cues [28].

5. Line 896: It is suggested that the authors elaborate further on how conducting FGDs with fewer than optimal numbers of participants may have affected the quality of the study, for example a narrower range of experiences may have been captured, limitations in the transferability of findings, and other impacts extending beyond participant interaction alone. This is a major limitation of this study.

**Response:** We thank the reviewer for this important observation and agree that conducting focus group discussions (FGDs) with fewer than optimal participant numbers constitutes a limitation of the study. Smaller group sizes may have restricted the range of experiences and perspectives captured within individual discussions and may limit the transferability of findings from those specific groups. In particular, FGDs with fewer participants may generate less variability in viewpoints and reduce opportunities for contrasting interpretations to emerge through group interaction.

At the same time, we considered whether exclusion of these FGDs would introduce other analytic or ethical limitations. Although methodological guidance commonly recommends larger groups, the qualitative methods literature also acknowledges that smaller FGDs can still generate meaningful interaction and data, particularly where participants engage actively and respond to one another, as described in some methodological accounts (e.g. Darshini et al., 2023). In our study, the FGD involving two participants nevertheless involved sustained discussion and mutual interaction rather than parallel individual accounts, and its content did not stand alone analytically but was interpreted in relation to patterns observed across multiple FGDs conducted with different group compositions.

Nonetheless, we recognise that findings from FGDs with fewer participants should be interpreted with appropriate caution. We have therefore revised the manuscript to more explicitly acknowledge that these smaller groups may have captured a narrower range of experiences and that this may affect the breadth and transferability of the findings, while also clarifying the rationale for retaining these data.

**Line 796-809**

In addition, some participants (n = 4) did not attend the focus group discussions (FGDs) for which they had registered, primarily due to scheduling difficulties. As a result, several FGDs were conducted with fewer than optimal numbers of participants, and in one case only two participants were present. Conducting FGDs with smaller group sizes may have restricted the range of experiences and perspectives captured within those discussions and may have limited opportunities for variability and contrasting interpretations to emerge through group interaction. Findings from these smaller groups should therefore be interpreted with appropriate caution, particularly with regard to breadth and transferability.

At the same time, methodological accounts in the qualitative literature acknowledge that smaller FGDs can still generate meaningful interaction and data where participants engage actively with one another for example [61]. In our study, the FGD involving two participants generated sustained discussion and mutual interaction rather than parallel individual accounts. Its content was not analysed in isolation but interpreted in relation to patterns observed across multiple FGDs conducted with different group compositions. Excluding these data was therefore considered to risk introducing other analytic and ethical limitations.

.
