# Supplementary material for: Navigating the unspoken: A qualitative study of employees’ perceptions of culture and norms surrounding sexual harassment at a Swedish university
Source: PLoS One. 2026 Jun 16;21(6):e0351724. doi: 10.1371/journal.pone.0351724 (PMC13271518; doi:10.1371/journal.pone.0351724)
Supplement: Peer review history

# Peer review: Decision Letter 0

**DOI:** 10.1371/journal.pone.0351724.r001

**Authors:** Kshitij Karki

2 Dec 2025

PONE-D-25-35396

Navigating the unspoken: a qualitative study of employees’ perceptions of culture and norms surrounding sexual harassment at a Swedish university

PLOS ONE

Dear Dr. Jack Wyndham Palmieri,

Thank you for submitting your manuscript to PLOS ONE. After careful consideration, we feel that it has merit but does not fully meet PLOS ONE’s publication criteria as it currently stands. Therefore, we invite you to submit a revised version of the manuscript that addresses the points raised during the review process.

Please submit your revised manuscript by Jan 16 2026 11:59PM. If you will need more time than this to complete your revisions, please reply to this message or contact the journal office at plosone@plos.org. When you're ready to submit your revision, log on to https://www.editorialmanager.com/pone/ and select the 'Submissions Needing Revision' folder to locate your manuscript file.

Please include the following items when submitting your revised manuscript:

- A rebuttal letter that responds to each point raised by the academic editor and reviewer(s). You should upload this letter as a separate file labeled 'Response to Reviewers'.
- A marked-up copy of your manuscript that highlights changes made to the original version. You should upload this as a separate file labeled 'Revised Manuscript with Track Changes'.
- An unmarked version of your revised paper without tracked changes. You should upload this as a separate file labeled 'Manuscript'.

If you would like to make changes to your financial disclosure, please include your updated statement in your cover letter. Guidelines for resubmitting your figure files are available below the reviewer comments at the end of this letter.

If applicable, we recommend that you deposit your laboratory protocols in protocols.io to enhance the reproducibility of your results. Protocols.io assigns your protocol its own identifier (DOI) so that it can be cited independently in the future. For instructions see: https://journals.plos.org/plosone/s/submission-guidelines#loc-laboratory-protocols. Additionally, PLOS ONE offers an option for publishing peer-reviewed Lab Protocol articles, which describe protocols hosted on protocols.io. Read more information on sharing protocols at https://plos.org/protocols?utm_medium=editorial-email&utm_source=authorletters&utm_campaign=protocols.

We look forward to receiving your revised manuscript.

Kind regards,

Kshitij Karki, MPH, MA

Academic Editor

PLOS ONE

Journal Requirements:

When submitting your revision, we need you to address these additional requirements.

1. Please ensure that your manuscript meets PLOS ONE's style requirements, including those for file naming. The PLOS ONE style templates can be found at

https://journals.plos.org/plosone/s/file?id=wjVg/PLOSOne_formatting_sample_main_body.pdf and

https://journals.plos.org/plosone/s/file?id=ba62/PLOSOne_formatting_sample_title_authors_affiliations.pdf.

2. Thank you for stating the following financial disclosure:

“This study was funded by the Swedish Research Council Grant number [2018-02457].”

Please state what role the funders took in the study.  If the funders had no role, please state: "The funders had no role in study design, data collection and analysis, decision to publish, or preparation of the manuscript."

If this statement is not correct you must amend it as needed.

Please include this amended Role of Funder statement in your cover letter; we will change the online submission form on your behalf.

3. We note that you have indicated that there are restrictions to data sharing for this study. For studies involving human research participant data or other sensitive data, we encourage authors to share de-identified or anonymized data. However, when data cannot be publicly shared for ethical reasons, we allow authors to make their data sets available upon request. For information on unacceptable data access restrictions, please see http://journals.plos.org/plosone/s/data-availability#loc-unacceptable-data-access-restrictions.

Before we proceed with your manuscript, please address the following prompts:

a) If there are ethical or legal restrictions on sharing a de-identified data set, please explain them in detail (e.g., data contain potentially identifying or sensitive patient information, data are owned by a third-party organization, etc.) and who has imposed them (e.g., a Research Ethics Committee or Institutional Review Board, etc.). Please also provide contact information for a data access committee, ethics committee, or other institutional body to which data requests may be sent.

b) If there are no restrictions, please upload the minimal anonymized data set necessary to replicate your study findings to a stable, public repository and provide us with the relevant URLs, DOIs, or accession numbers. Please see http://www.bmj.com/content/340/bmj.c181.long for guidelines on how to de-identify and prepare clinical data for publication. For a list of recommended repositories, please see https://journals.plos.org/plosone/s/recommended-repositories. You also have the option of uploading the data as Supporting Information files, but we would recommend depositing data directly to a data repository if possible.

4. If the reviewer comments include a recommendation to cite specific previously published works, please review and evaluate these publications to determine whether they are relevant and should be cited. There is no requirement to cite these works unless the editor has indicated otherwise.

Additional Editor Comments (if provided):

Please respond to the feedback from the reviewers. Thank you

[Note: HTML markup is below. Please do not edit.]

Reviewers' comments:

Reviewer's Responses to Questions

**Comments to the Author**

1. Is the manuscript technically sound, and do the data support the conclusions?

The manuscript must describe a technically sound piece of scientific research with data that supports the conclusions. Experiments must have been conducted rigorously, with appropriate controls, replication, and sample sizes. The conclusions must be drawn appropriately based on the data presented.

Reviewer #1: Partly

Reviewer #2: Partly

Reviewer #3: Yes

**********

2. Has the statistical analysis been performed appropriately and rigorously?

Reviewer #1: N/A

Reviewer #2: No

Reviewer #3: Yes

**********

3. Have the authors made all data underlying the findings in their manuscript fully available?

The PLOS Data policy requires authors to make all data underlying the findings described in their manuscript fully available without restriction, with rare exception (please refer to the Data Availability Statement in the manuscript PDF file). The data should be provided as part of the manuscript or its supporting information, or deposited to a public repository. For example, in addition to summary statistics, the data points behind means, medians and variance measures should be available. If there are restrictions on publicly sharing data—e.g. participant privacy or use of data from a third party—those must be specified.

Reviewer #1: No

Reviewer #2: No

Reviewer #3: Yes

**********

4. Is the manuscript presented in an intelligible fashion and written in standard English?

PLOS ONE does not copyedit accepted manuscripts, so the language in submitted articles must be clear, correct, and unambiguous. Any typographical or grammatical errors should be corrected at revision, so please note any specific errors here.

Reviewer #1: No

Reviewer #2: No

Reviewer #3: Yes

**********

5. Review Comments to the Author

Please use the space provided to explain your answers to the questions above. You may also include additional comments for the author, including concerns about dual publication, research ethics, or publication ethics. (Please upload your review as an attachment if it exceeds 20,000 characters)

Reviewer #1: Thank you, PLOS ONE for the opportunity to review this manuscript reporting employees’ perceptions of culture and norms surrounding sexual harassment at a Swedish university. Please find my comments and recommendations below.

1. Kindly suggesting reducing the word count, the manuscript is a bit lengthy and there is room for concise communication throughout the manuscript to enhance readability and brevity.

2. The introduction can be strengthened by maintaining a flow of ideas that are linked from one paragraph to the next, clearly articulating the problem being researched. Currently the introduction seems quite long with ideas that are not flowing so well together. Suggesting framing the introduction in this manner. Firstly, providing background context-clearly describing the situation that gives rise to the problem. Secondly, clearly stating the problem that exist. Thirdly, explaining why the problem matters-the consequences if the problem is not addressed. Fourthly, indicating the knowledge gap and how your research will help fill the gap or solve the problem.

3. Line 24: Define “recent”-if possible, provide the dates of the meta-analyses for better understanding of when the meta-analyses were done.

4. Line 75: Please provide reference for this statement.

5. Line 75: Kindly elaborate the multiple countries, are they from the same continent or different continents? European, Asian etc

6. Line 90: Define “recent”-if possible, provide the dates of the systematic review for better understanding of when the review was done.

7. Line 105: Put an apostrophe symbol on employees (employees’)

8. Line 112: Remove current

9. Line 112-113: Indicate that this is a qualitative study.

Methods

10. Line 179-196: Kindly move Ethical Considerations to be the first paragraph in the methods section.

11. Please add a subheading study population, location and sampling before data collection.

a. Add the following points under this topic

-Defining eligibility criteria for participation in the FGDs

-Study locations where FGDs were held

-Participants sampling (was it purposive, random etc)

-Description of the each FGD participants (how many in each group, the gender composition, was it mixed departments in each FGD)

12. Line 124-141: This belongs in the introduction section as it is providing background information which is helpful to the international audience.

13. Line 140: Kindly elaborate on the key findings: what was the prevalence of SH, what were the characteristics of SH, does these key findings provide justification of the research problem.

14. Line 142: The use of the word current study is sort of confusing, does this imply that the study is still ongoing?

15. Please clarify the criteria for participation in the study so that it becomes clear. As it reads now, what does all employees mean. Is it all university employees, is it employees from a specific department? This falls under study population, location, sampling subheading.

16. Line 142-143: Belongs in the study population, location, sampling subheading. Please elaborate more on the recruitment, where the posters e-posters, where were they placed at the university? How many people expressed interest to participate in the FGDs? Did everyone who expressed interest end up participating in the FGDs, if not how was sampling done for the participants who participated.

17. Line 148: Kindly provide reference for the guide, is it in Appendix?

18. Line 157: Kindly provide clarity on the justification for having 10 FGDs with some FGDs having 2, 3 participants. Were all these FGDs conducted at Lund University, was it not possible to have less FGD with a reasonable number of participants in each FGD. How did you select the participant in each FGD?

19. Line 166: Table 1, participant characteristics are usually presented under the Results sections as the first point describing the key demographics of the participants. Kindly provide the age ranges of the participants. Other than the sex and language did you collect any other demographics information for the participants?

20. Line 166: Table 1, FGD 8 only had 2 participants. Ideally an FGD leverages on the group dynamics and interpersonal interactions among multiple people in-order to generate broad range of ideas. I am not sure if this was achieved by having only 2 participants, could you provide justification for conducting an FGD with only 2 people.

21. Line 168, who transcribed the audio recordings? The audio recording in Swedish where they translated to English? Kindly elaborate.

22. Line 195: Were the audio-records deleted from the audio-recorders?

23. Line 181: You have already spelt out the acronym in line 145.

Results

24. Line 200: Suggesting presenting the sentence in this manner; ‘…that presents perceptions of Swedish university employees on the organisational culture and workplace norms surrounding SH.’ Themes are showing us what they think about SH.

25. The results section is a bit difficult to follow, the presented themes are a bit broad and more descriptive rather than analytical.

26. Line 216: Is there supposed to be a Figure 1 inserted here?

27. Line 218: Kindly rephrase, following section implies you are moving from the results section. Maybe say following paragraphs.

28. There is mention of the #MeToo a couple of times in the results and discussion section, however in the introduction you have not provided much background on what it is about.

29. Line 364: What is an Ostrich effect?

30. Line 376: What is ‘Scandinavian complacency’? Its good practice to explain phrases in a manuscript as some readers may not be familiar with them.

31. Line 443: Put a full-stop after the sentence.

Discussion

32. Line 635: Provide the dates of the meta-analysis and the author.

33. In the discussion section, suggesting you focus on the key issues coming out of the results section and mapping the way forward rather than discussing all issues presented in the results sections.

34. Line 767: This section is the study strengths and limitations, present it as such rather than methodological considerations.

35. Line 789: You start by saying other limitations, but you have not mentioned any limitations for the study.

Reviewer #2: dear authors,

-it is important to take into account both content and thematic analysis. So Granheim alone does not suffice.

-Clarify the qualitative study more- can it fit as an interpretive phenomenological analysis?

-Study data cannot be shared ethically , but interview transcripts can be uploaded in the repository

-IRB letter can also be uploaded- from Malmo and Lund university

-How was the data analyzed?

-A different method, such as a Delphi group for example, would be more

appropriate than IPA to seek agreement among experts

-It is important to give more demographic profiling

-please follow APA guide as to how to write experiential and exponential statements when reporting interviewed statements

-references need to be updated,

-out of context references are mentioned, e.g 13- harassments in Indian context.

Reviewer #3: I have thoroughly reviewed your manuscript and commend its valuable contribution to understanding sexual harassment among employees in tertiary institutions. It is well written, with a background that effectively situates the issue within both global and local contexts. The problem is clearly defined, and the research questions are well articulated and aligned with the central issue under investigation.

The study methodology is clearly articulated, with an appropriate design, sampling strategy, and data collection methods that align well with the research problem under investigation. The discussion is well developed, highlighting the significance of the study and contextualising the findings while drawing meaningful comparisons with previous research both globally and locally.

The strengths and limitations of the study are clearly articulated and the ways in which these were addressed are well explained. The implications of the findings for managing sexual harassment among employees in tertiary institutions are thoughtfully discussed, along with considerations for future policies and interventions. However, here are a few minor comments on aspects that deserve your attention to further improve the article.

Methods

Lines 124-141: Please separate sample/sampling procedures from data collection procedures by creating separate subheadings under methods section. This makes it easier for readers to follow.

Lines 153-154: Suggesting that you briefly describe Malmö University, where it is located, student enrolment and staff; this helps readers understand how the pilot university resembles the university under study.

Results

Include data collection timelines: when was data collected?

Did you have international staff/employees participating in the group discussions? How many? If yes you could include this under participant characteristics.

**********

6. PLOS authors have the option to publish the peer review history of their article (what does this mean?). If published, this will include your full peer review and any attached files.

If you choose “no”, your identity will remain anonymous but your review may still be made public.

**Do you want your identity to be public for this peer review?**  For information about this choice, including consent withdrawal, please see our Privacy Policy.

Reviewer #1: No

Reviewer #2: **Yes:** yumna ali

Reviewer #3: No

**********

[NOTE: If reviewer comments were submitted as an attachment file, they will be attached to this email and accessible via the submission site. Please log into your account, locate the manuscript record, and check for the action link "View Attachments". If this link does not appear, there are no attachment files.]

To ensure your figures meet our technical requirements, please review our figure guidelines: https://journals.plos.org/plosone/s/figures

You may also use PLOS’s free figure tool, NAAS, to help you prepare publication quality figures: https://journals.plos.org/plosone/s/figures#loc-tools-for-figure-preparation.

NAAS will assess whether your figures meet our technical requirements by comparing each figure against our figure specifications.

# Author comment: Author response to Decision Letter 1

**DOI:** 10.1371/journal.pone.0351724.r002

31 Mar 2026

Authors’ response to editor and reviewer’s comments

Title: Navigating the unspoken: a qualitative study of employees’ perceptions of culture and norms surrounding sexual harassment at a Swedish university

Author names: Jack Palmieri, Frida Pilgaard, Anette Agardh

Version: 2

Date: 2026-03-24

Authors’ response to reviewers: See the following pages.

Dear Kshitij Karki,

We would like to thank you and the reviewer(s) for the valuable feedback. We have revised the manuscript in line with this feedback. Please see the revised version attached and our responses to the reviewers’ comments, point by point, below.

With kind regards,

Jack Palmieri

Comments from Reviewer #1

1. Thank you, PLOS ONE for the opportunity to review this manuscript reporting employees’ perceptions of culture and norms surrounding sexual harassment at a Swedish university. Please find my comments and recommendations below.

Response: We thank the reviewer for their time and efforts.

2. Kindly suggesting reducing the word count, the manuscript is a bit lengthy and there is room for concise communication throughout the manuscript to enhance readability and brevity.

Response: We thank the reviewer for this comment. We have shortened the manuscript in a number of areas, most significantly in the introduction section.

3. The introduction can be strengthened by maintaining a flow of ideas that are linked from one paragraph to the next, clearly articulating the problem being researched. Currently the introduction seems quite long with ideas that are not flowing so well together. Suggesting framing the introduction in this manner. Firstly, providing background context-clearly describing the situation that gives rise to the problem. Secondly, clearly stating the problem that exist. Thirdly, explaining why the problem matters-the consequences if the problem is not addressed. Fourthly, indicating the knowledge gap and how your research will help fill the gap or solve the problem.

Response: We thank the reviewer for this comment. We have streamlined the introduction both for brevity and flow of ideas as you suggest.

4. Line 24: Define “recent”-if possible, provide the dates of the meta-analyses for better understanding of when the meta-analyses were done.

Response: We thank the reviewer for this comment. The meta-analyses in question were by Latcheva from 2017 and Debenath 2025. This information has now been added to the manuscript.

Lines 42 and 43

With recent meta-analyses by Latcheva (2017) and Debnath (2025) confirming that it remains a serious and under-addressed form of gender-based violence [2, 3]

5. Line 75: Please provide reference for this statement.

Response: We thank the reviewer for this comment. The correct reference has now been added.

6. Line 75: Kindly elaborate the multiple countries, are they from the same continent or different continents? European, Asian etc

Response: We thank the reviewer for this clarification. The cited reference was conducted in Belgium, Croatia, Germany, and Sweden. This has been clarified in the manuscript in the following way

In European settings, uncertainty regarding definitions, reporting channels, and access to support has been shown to impact reporting and prevention

7. Line 90: Define “recent”-if possible, provide the dates of the systematic review for better understanding of when the review was done.

Response: Thank you for this suggestion. The systematic review dates from 2024. This has now been added to the manuscript as follows

A 2024 systematic review of gender-based violence in academia found that over half of university staff had experienced some form of interpersonal violence, with women and LGBTQ+ staff reporting disproportionate exposure to SH, stalled career advancement, and reduced psychological well-being [14]

8. Line 105: Put an apostrophe symbol on employees (employees’)

Response: We thank the reviewer for this; the apostrophe has been added.

9. Line 112: Remove current

Response: We thank the reviewer for this, current has been removed, and the sentence reformatted as follows:

In order to fill these knowledge gaps, this study aimed to explore how employees at a Swedish university perceive the organisational culture and workplace norms surrounding sexual harassment.

10. Line 112-113: Indicate that this is a qualitative study.

Response: We thank the reviewer for this, the term qualitative has been added as follows:

To address these gaps, this qualitative study aimed to explore how employees at a Swedish university perceive the organisational culture and workplace norms surrounding sexual harassment.

Methods

11. Line 179-196: Kindly move Ethical Considerations to be the first paragraph in the methods section.

Response: We thank the reviewer for this suggestion. As several elements discussed in ethical considerations relate to information first introduced in the methods section, however, we have chosen to keep the ethical considerations section where it is in the manuscript to enhance flow and improve readability.

12. Please add a subheading study population, location and sampling before data collection.

a. Add the following points under this topic

-Defining eligibility criteria for participation in the FGDs

-Study locations where FGDs were held

-Participants sampling (was it purposive, random etc)

-Description of the each FGD participants (how many in each group, the gender composition, was it mixed departments in each FGD)

Response: We thank the reviewer for the comment. We have now added a section named study population and sampling. In this section we have described the study setting, clarified the eligibility criteria and sampling. Information regarding how the focus groups were comprised has been clarified but kept under data collection.

13. Line 124-141: This belongs in the introduction section as it is providing background information which is helpful to the international audience.

Response: We thank the reviewer for this suggestion. We agree that information regarding the university and its students and staff are important for any audience. However, we see study setting as being the most relevant section for this as it pertains to the specifics of this study rather than the broad background of the topic. However, the section has been shortened and moved to study setting and, in line with your comment 14, key figures have been added to the introduction.

14. Line 140: Kindly elaborate on the key findings: what was the prevalence of SH, what were the characteristics of SH, does these key findings provide justification of the research problem.

Response: We thank the reviewer for this suggestion. These key findings have been elaborated upon in the introduction in line with comment 13.

In the Swedish academic context, a 2019 survey of staff at conducted as part of the Tellus project at Lund University showed that 25% of female employees and 7% of male employees report experiences of SH during their employment [19]. When limiting the response period to the past 12 months, the corresponding figures were around 8% of female employees, and 3% of male employees [19].

15. Line 142: The use of the word current study is sort of confusing, does this imply that the study is still ongoing?

Response: We thank the reviewer for this comment. The word current has been removed from this sentence.

16. Please clarify the criteria for participation in the study so that it becomes clear. As it reads now, what does all employees mean. Is it all university employees, is it employees from a specific department? This falls under study population, location, sampling subheading.

Response: We thank the reviewer for this comment. Participation was open to all employees of the university regardless of department, position, type of employment or experience of SH. The information has been added to the new section as suggested.

17. Line 142-143: Belongs in the study population, location, sampling subheading. Please elaborate more on the recruitment, where the posters e-posters, where were they placed at the university? How many people expressed interest to participate in the FGDs? Did everyone who expressed interest end up participating in the FGDs, if not how was sampling done for the participants who participated.

Response: We thank the reviewer for this comment. Posters were physical and displayed in all faculties of the university. Internal communication channels including email was used to distribute the information to all employees using university email lists. All respondents who expressed interest in participating were offered a place in an FGD. Not all participants who signed up for an FGD attended. This information has been added to the manuscript

18. Line 148: Kindly provide reference for the guide, is it in Appendix?

Response: We thank the reviewer for this suggestion. The FGD guide has been added as an appendix.

19. Line 157: Kindly provide clarity on the justification for having 10 FGDs with some FGDs having 2, 3 participants. Were all these FGDs conducted at Lund University, was it not possible to have less FGD with a reasonable number of participants in each FGD. How did you select the participant in each FGD?

Response: We thank the reviewer for this comment. Following the recommendations in guiding literature, and our own experiences of booking and conducting FGDs, our aim was to conduct FGDs with 5-8 participants in each. Practical considerations, however, regarding booking of participants, finding time slots that worked, and ensuring those with managerial responsibility were separated from those without, led to some FGDs being booked with 4-6 participants. In a number of cases, participants did not turn up (n=4), and the FGDs were therefore conducted with the numbers shown in the manuscript. This was of course regrettable but considered preferable to cancelling the FGD at the last minute. This issue has now been clarified in the strengths and limitations in the Discussion.

In addition, some participants (n=4) did not attend the FGDs they had booked. These participants primarily gave scheduling challenges as the reason, but the result was that several FGDs were conducted with less than optimal numbers of participants.

20. Line 166: Table 1, participant characteristics are usually presented under the Results sections as the first point describing the key demographics of the participants. Kindly provide the age ranges of the participants. Other than the sex and language did you collect any other demographics information for the participants?

Response: We thank the reviewer for this suggestion. Participant characteristics have been moved to the results section. No demographic data were collected on the participants beyond what is already presented here. This was a deliberate strategy to further protect the identities of the participants considering the potentially sensitive nature of the questions.

21. Line 166: Table 1, FGD 8 only had 2 participants. Ideally an FGD leverages on the group dynamics and interpersonal interactions among multiple people in-order to generate broad range of ideas. I am not sure if this was achieved by having only 2 participants, could you provide justification for conducting an FGD with only 2 people.

Response: We thank the reviewer for this and agree that FGDs must always leverage the interaction between participants. As explained in answer 19, no FGDs were planned with 2 (or even 3) participants, but the decision was made to continue with these even when participants did not attend. The facilitator is experienced in conducting FGDs and felt that the information collected during this FGD was equally valuable to include in the analysis.

22. Line 168, who transcribed the audio recordings? The audio recording in Swedish where they translated to English? Kindly elaborate.

Response: The audio recordings were transcribed verbatim in Swedish by a research assistant. Translation to English took place during the coding process by the first author. This has also been clarified in the text as below.

All FGDs were audio recorded and transcribed verbatim by a research assistant at the university. The analysis followed the principles of qualitative content analysis according to Graneheim and Lundman [20]. Firstly, all transcripts were read through multiple times to gain familiarity with the data. Following this, meaning units relevant to the research aim were identified and coded inductively in English by the first author. These codes were then grouped into sub-categories and categories through a combination of inductive and deductive processes, moving iteratively between the empirical data and emerging interpretations.

Categories were subsequently examined for the development of latent sub-themes through a process of pattern recognition and conceptual reflection. Finally, one overarching theme was formulated. Quotes were selected to illustrate the categories and these were translated to English by the first author. The final model was developed collaboratively, with all co-authors reviewing coding decisions and contributing to analytical refinement.

23. Line 195: Were the audio-records deleted from the audio-recorders?

Response: All recordings were deleted from the recorders immediately after they had been transferred to the encrypted hard drives which were kept locked in a secure safe at the university. This has been clarified in the manuscript.

All identifying information was removed during the transcription process to protect participant anonymity. All audio recordings were transferred to encrypted hard drives immediately after the FGD and deleted from the audio recorders. These audio recordings and transcripts were securely stored on encrypted drives with access restricted to members of the research team. Consent forms were stored separately in a locked safe.

24. Line 181: You have already spelt out the acronym in line 145.

Response: Thank you for noting this – this has now been rectified in the manuscript.

Results

25. Line 200: Suggesting presenting the sentence in this manner; ‘…that presents perceptions of Swedish university employees on the organisational culture and workplace norms surrounding SH.’ Themes are showing us what they think about SH.

Response: We thank the reviewer for this suggestion. We have re-written this sentence as follows.

The analysis of the focus group discussions resulted in one overarching theme, four sub-themes, and eight categories that reflect how Swedish university employees perceive the organisational norms and workplace culture surrounding

26. The results section is a bit difficult to follow, the presented themes are a bit broad and more descriptive rather than analytical.

Response: We thank the reviewer for this feedback. However, we feel that the presented results are an accurate reflection of the analysis process and represent the correct level of abstraction and interpretation.

27. Line 216: Is there supposed to be a Figure 1 inserted here?

Response: We thank the reviewer for this comment. Following the submission guidelines for PLOS One we have not put any of the figures in the manuscript file but rather submitted them as separate images. Hence the placeholders and heading are left, but the figures themselves are in a separate file.

28. Line 218: Kindly rephrase, following section implies you are moving from the results section. Maybe say following paragraphs.

Response: We thank the reviewer for this comment. We have changed this in the l

Text to “following paragraphs”

29. There is mention of the #MeToo a couple of times in the results and discussion section, however in the introduction you have not provided much background on what it is about.

Response: We thank the reviewer for this feedback. We feel that the #MeToo campaign was sufficiently global to not require significant description. To support readers who may not be acquainted with the movement, however, a reference has been added in the introduction as follows.

Denmark found that employees often struggled to label their experiences as SH, a process shaped by peer responses, cultural dis

Attachment Submitted filename: Response to Reviewers Navigating the unspoken Final.docx

# Peer review: Decision Letter 1

**DOI:** 10.1371/journal.pone.0351724.r003

**Authors:** Kshitij Karki

23 Apr 2026

PONE-D-25-35396R1

Navigating the unspoken: a qualitative study of employees’ perceptions of culture and norms surrounding sexual harassment at a Swedish university

PLOS One

Dear Dr. Palmieri,

Thank you for submitting your manuscript to PLOS ONE. After careful consideration, we feel that it has merit but does not fully meet PLOS ONE’s publication criteria as it currently stands. Therefore, we invite you to submit a revised version of the manuscript that addresses the points raised during the review process.

Please address the comments from the reviewers.

Please submit your revised manuscript by Jun 07 2026 11:59PM. If you will need more time than this to complete your revisions, please reply to this message or contact the journal office at plosone@plos.org. When you're ready to submit your revision, log on to https://www.editorialmanager.com/pone/ and select the 'Submissions Needing Revision' folder to locate your manuscript file.

Please include the following items when submitting your revised manuscript:

- A letter that responds to each point raised by the academic editor and reviewer(s). You should upload this letter as a separate file labeled 'Response to Reviewers'.
- A marked-up copy of your manuscript that highlights changes made to the original version. You should upload this as a separate file labeled 'Revised Manuscript with Track Changes'.
- An unmarked version of your revised paper without tracked changes. You should upload this as a separate file labeled 'Manuscript'.

If you would like to make changes to your financial disclosure, please include your updated statement in your cover letter. Guidelines for resubmitting your figure files are available below the reviewer comments at the end of this letter.

If applicable, we recommend that you deposit your laboratory protocols in protocols.io to enhance the reproducibility of your results. Protocols.io assigns your protocol its own identifier (DOI) so that it can be cited independently in the future. For instructions see: https://journals.plos.org/plosone/s/submission-guidelines#loc-laboratory-protocols. Additionally, PLOS ONE offers an option for publishing peer-reviewed Lab Protocol articles, which describe protocols hosted on protocols.io. Read more information on sharing protocols at https://plos.org/protocols?utm_medium=editorial-email&utm_source=authorletters&utm_campaign=protocols.

As the corresponding author, your ORCID iD is verified in the submission system and will appear in the published article. PLOS supports the use of ORCID, and we encourage all coauthors to register for an ORCID iD and use it as well. Please encourage your coauthors to verify their ORCID iD within the submission system before final acceptance, as unverified ORCID iDs will not appear in the published article. *Only*  the individual author can complete the verification step; PLOS staff *cannot*  verify ORCID iDs on behalf of authors.

We look forward to receiving your revised manuscript.

Kind regards,

Kshitij Karki, MPH, MA

Academic Editor

PLOS One

**Journal Requirements:**

1. If the reviewer comments include a recommendation to cite specific previously published works, please review and evaluate these publications to determine whether they are relevant and should be cited. There is no requirement to cite these works unless the editor has indicated otherwise.

2. Please review your reference list to ensure that it is complete and correct. If you have cited papers that have been retracted, please include the rationale for doing so in the manuscript text, or remove these references and replace them with relevant current references. Any changes to the reference list should be mentioned in the rebuttal letter that accompanies your revised manuscript. If you need to cite a retracted article, indicate the article’s retracted status in the References list and also include a citation and full reference for the retraction notice.

[Note: HTML markup is below. Please do not edit.]

Reviewers' comments:

Reviewer's Responses to Questions

**Comments to the Author**

1. If the authors have adequately addressed your comments raised in a previous round of review and you feel that this manuscript is now acceptable for publication, you may indicate that here to bypass the “Comments to the Author” section, enter your conflict of interest statement in the “Confidential to Editor” section, and submit your "Accept" recommendation.

Reviewer #1: All comments have been addressed

Reviewer #2: All comments have been addressed

Reviewer #3: (No Response)

**********

2. Is the manuscript technically sound, and do the data support the conclusions?

The manuscript must describe a technically sound piece of scientific research with data that supports the conclusions. Experiments must have been conducted rigorously, with appropriate controls, replication, and sample sizes. The conclusions must be drawn appropriately based on the data presented.

Reviewer #1: Yes

Reviewer #2: Partly

Reviewer #3: Yes

**********

3. Has the statistical analysis been performed appropriately and rigorously?

Reviewer #1: N/A

Reviewer #2: No

Reviewer #3: N/A

**********

4. Have the authors made all data underlying the findings in their manuscript fully available?

The PLOS Data policy requires authors to make all data underlying the findings described in their manuscript fully available without restriction, with rare exception (please refer to the Data Availability Statement in the manuscript PDF file). The data should be provided as part of the manuscript or its supporting information, or deposited to a public repository. For example, in addition to summary statistics, the data points behind means, medians and variance measures should be available. If there are restrictions on publicly sharing data—e.g. participant privacy or use of data from a third party—those must be specified.

Reviewer #1: Yes

Reviewer #2: No

Reviewer #3: Yes

**********

5. Is the manuscript presented in an intelligible fashion and written in standard English?

PLOS ONE does not copyedit accepted manuscripts, so the language in submitted articles must be clear, correct, and unambiguous. Any typographical or grammatical errors should be corrected at revision, so please note any specific errors here.

Reviewer #1: Yes

Reviewer #2: Yes

Reviewer #3: Yes

**********

6. Review Comments to the Author

Please use the space provided to explain your answers to the questions above. You may also include additional comments for the author, including concerns about dual publication, research ethics, or publication ethics. (Please upload your review as an attachment if it exceeds 20,000 characters)

Reviewer #1: (No Response)

Reviewer #2: Dear authors,

Most of the comments were met, but the Delphi approach would have improved the study. Please update the literature. Also upload transcripts and related data in repository.

Please explicitly mention which software was used to group data in themes and codes.

Reviewer #3: (No Response)

**********

7. PLOS authors have the option to publish the peer review history of their article (what does this mean?). If published, this will include your full peer review and any attached files.

If you choose “no”, your identity will remain anonymous but your review may still be made public.

**Do you want your identity to be public for this peer review?**  For information about this choice, including consent withdrawal, please see our Privacy Policy.

Reviewer #1: No

Reviewer #2: **Yes:** yumna ali

Reviewer #3: No

**********

[NOTE: If reviewer comments were submitted as an attachment file, they will be attached to this email and accessible via the submission site. Please log into your account, locate the manuscript record, and check for the action link "View Attachments". If this link does not appear, there are no attachment files.]

To ensure your figures meet our technical requirements, please review our figure guidelines: https://journals.plos.org/plosone/s/figures

You may also use PLOS’s free figure tool, NAAS, to help you prepare publication quality figures: https://journals.plos.org/plosone/s/figures#loc-tools-for-figure-preparation.

NAAS will assess whether your figures meet our technical requirements by comparing each figure against our figure specifications.

Attachment Submitted filename: Reviewer 3 comments PONE-D-25-35396R1.docx

# Author comment: Author response to Decision Letter 2

**DOI:** 10.1371/journal.pone.0351724.r004

6 May 2026

Authors’ response to editor and reviewer’s comments

Title: Navigating the unspoken: a qualitative study of employees’ perceptions of culture and norms surrounding sexual harassment at a Swedish university

Author names: Jack Palmieri, Frida Pilgaard, Anette Agardh

Version: 3

Date: 2026-05-06

Authors’ response to reviewers: See the following pages.

Dear Kshitij Karki,

We would like to thank you and the reviewers for the valuable feedback once again. We have revised the manuscript in line with this feedback. Please see the revised version attached and our responses to the reviewers’ comments, point by point, below.

With kind regards,

Jack Palmieri

Comments from Reviewer #1

1. No Further comments

Comments from Reviewer #2

1. Most of the comments were met, but the Delphi approach would have improved the study.

Response: We thank the reviewer for this suggestion. We agree that the Delphi approach is a powerful method for achieving expert consensus. Its focus on iteration, control and expert opinion make it an appropriate method to use for certain research questions. The aim of the present study, however, was to explore shared perceptions of organisational culture and workplace norms surrounding sexual harassment among employees, rather than to seek expert agreement or consensus. The study builds on existing research that captures the discursive and collaborative nature of culture and norms.

The study therefore used focus group discussions, which emphasise interaction, dialogue, and collective meaning-making, elements that are central to understanding organisational culture. Participants were not recruited as experts, and consensus was not an analytic goal. For these reasons, a Delphi approach was not considered methodologically appropriate for this study.

2. Please update the literature.

Response: We thank the reviewer for this comment. We have reviewed the literature list, updated references where needed (including adding DOIs where possible) and added two additional references, one of which has been published since the reviewing process began. More specifically these references draw on the normalisation of harassment by male students in South Africa, and the related concept of “dangerous common sense” in the same context (Kiguwa et al., 2015; Jagath & Hamlall, 2024). These have been added to the discussion as follows:

Lines 678-682

This process of collectively negotiated meaning‑making echoes findings from studies from South African Universities, where focus group‑based research among students and staff has shown how everyday verbal and physical violations are frequently framed as ordinary expressions of masculinity or institutional life (“dangerous common sense”), rendering them unremarkable and difficult to contest [42, 43]

3. Also upload transcripts and related data in repository.

Response: We thank the reviewer for this suggestion. In this case The interview transcripts and associated personal data cannot be made publicly available because they were collected from a small, potentially identifiable group and contain sensitive information. Participants did not consent to sharing their de-identified data beyond the research team, and doing so would conflict with the terms of our ethical approval. Access to the underlying data is therefore restricted in accordance with Swedish legal requirements and the conditions set by the Swedish Ethical Review Authority. The data are securely deposited at Lund University (Sweden). Data requests may be made to Lund University via registrator@lu.se and will be subject to review and approval by the Ethical Review Authority.

4. Please explicitly mention which software was used to group data in themes and codes.

Response: We thank the reviewer for this comment. Data coding and analysis was conducted using OpenCode 4. This has now been added to the manuscript as follows.

Lines 161-162

Data analysis was conducted using OpenCode 4 [26].

Comments from Reviewer #3

1. Thank you for sharing this revised manuscript for further review and comments. I would like to appreciate the authors’ efforts in revising this manuscript in line with reviewer comments. While authors have adequately addressed most comments raised by reviewers, I bring their attention to a few comments below:

Response: We thank the reviewer for their kind feedback and for their continued efforts to improve the manuscript.

2. Check punctuation through the whole manuscript, for example:

a. Line 51: Place a comma after to: […] to organisational climate.

b. Line 54-56: Place a comma after SH […] not only individual incidents of SH but also the wider organisational norms […]

c. Line 57-58: Place a comma after challenged: […]. whether SH is acknowledged, challenged, or normalised.

Response: We thank the reviewer for these comments. We have reviewed the punctuation throughout the manuscript and made minor corrections where appropriate. The manuscript follows UK English grammar and punctuation conventions. Having considered the specific examples raised, we believe that the suggested commas are not required under standard UK usage and, in these cases, do not improve clarity. We have therefore not made changes to those instances.

Discussion:

3. Address capitalisation for consistency across headings through the whole manuscript e.g., line 563: Facing a culture of silence and normalisation or line 707: Negotiating Collective Norms and Meanings.

Response: We thank the reviewer for this valuable feedback. We have now standardised capitalisation using sentence case to reflect the journal requirements.

For example:

Line 164: Ethical considerations

Line 605: Negotiating collective norms and meanings

Line 688: Navigating power relations and organisational management

Line 727: Facing a culture of silence and normalisation

4. Line 714: Suggesting you state who coined the Classic Symbolic Interactionist Theory and year.

Response: We thank the reviewer for this response and agree that it strengthens the text. We have added the information as follows:

Line 613

Seeing the boundary of what constitutes SH as a negotiated reality mirrors what classic symbolic interactionist theory (coined by Herbert Blumer in 1937 [27] ) terms a negotiated order, an interpersonal process in which actors continuously redefine situations through talk, eye-contact and tacit cues [28].

5. Line 896: It is suggested that the authors elaborate further on how conducting FGDs with fewer than optimal numbers of participants may have affected the quality of the study, for example a narrower range of experiences may have been captured, limitations in the transferability of findings, and other impacts extending beyond participant interaction alone. This is a major limitation of this study.

Response: We thank the reviewer for this important observation and agree that conducting focus group discussions (FGDs) with fewer than optimal participant numbers constitutes a limitation of the study. Smaller group sizes may have restricted the range of experiences and perspectives captured within individual discussions and may limit the transferability of findings from those specific groups. In particular, FGDs with fewer participants may generate less variability in viewpoints and reduce opportunities for contrasting interpretations to emerge through group interaction.

At the same time, we considered whether exclusion of these FGDs would introduce other analytic or ethical limitations. Although methodological guidance commonly recommends larger groups, the qualitative methods literature also acknowledges that smaller FGDs can still generate meaningful interaction and data, particularly where participants engage actively and respond to one another, as described in some methodological accounts (e.g. Darshini et al., 2023). In our study, the FGD involving two participants nevertheless involved sustained discussion and mutual interaction rather than parallel individual accounts, and its content did not stand alone analytically but was interpreted in relation to patterns observed across multiple FGDs conducted with different group compositions.

Nonetheless, we recognise that findings from FGDs with fewer participants should be interpreted with appropriate caution. We have therefore revised the manuscript to more explicitly acknowledge that these smaller groups may have captured a narrower range of experiences and that this may affect the breadth and transferability of the findings, while also clarifying the rationale for retaining these data.

Line 796-809

In addition, some participants (n = 4) did not attend the focus group discussions (FGDs) for which they had registered, primarily due to scheduling difficulties. As a result, several FGDs were conducted with fewer than optimal numbers of participants, and in one case only two participants were present. Conducting FGDs with smaller group sizes may have restricted the range of experiences and perspectives captured within those discussions and may have limited opportunities for variability and contrasting interpretations to emerge through group interaction. Findings from these smaller groups should therefore be interpreted with appropriate caution, particularly with regard to breadth and transferability.

At the same time, methodological accounts in the qualitative literature acknowledge that smaller FGDs can still generate meaningful interaction and data where participants engage actively with one another for example [61]. In our study, the FGD involving two participants generated sustained discussion and mutual interaction rather than parallel individual accounts. Its content was not analysed in isolation but interpreted in relation to patterns observed across multiple FGDs conducted with different group compositions. Excluding these data was therefore considered to risk introducing other analytic and ethical limitations.

Attachment Submitted filename: Response to Reviewers Navigating the unspoken_Review 2.docx

# Peer review: Decision Letter 2

**DOI:** 10.1371/journal.pone.0351724.r005

**Authors:** Kshitij Karki

2 Jun 2026

Navigating the unspoken: a qualitative study of employees’ perceptions of culture and norms surrounding sexual harassment at a Swedish university

PONE-D-25-35396R2

Dear Dr. Jack Wyndham Palmieri,

We’re pleased to inform you that your manuscript has been judged scientifically suitable for publication and will be formally accepted for publication once it meets all outstanding technical requirements.

Within one week, you’ll receive an e-mail detailing the required amendments. When these have been addressed, you’ll receive a formal acceptance letter and your manuscript will be scheduled for publication.

An invoice will be generated when your article is formally accepted. Please note, if your institution has a publishing partnership with PLOS and your article meets the relevant criteria, all or part of your publication costs will be covered. Please make sure your user information is up-to-date by logging into Editorial Manager at Editorial Manager® and clicking the ‘Update My Information' link at the top of the page. For questions related to billing, please contact billing support.

If your institution or institutions have a press office, please notify them about your upcoming paper to help maximize its impact. If they’ll be preparing press materials, please inform our press team as soon as possible -- no later than 48 hours after receiving the formal acceptance. Your manuscript will remain under strict press embargo until 2 pm Eastern Time on the date of publication. For more information, please contact onepress@plos.org.

Kind regards,

Kshitij Karki, MPH, MA

Academic Editor

PLOS One

Additional Editor Comments (optional):

Reviewers' comments:

Reviewer's Responses to Questions

**Comments to the Author**

1. If the authors have adequately addressed your comments raised in a previous round of review and you feel that this manuscript is now acceptable for publication, you may indicate that here to bypass the “Comments to the Author” section, enter your conflict of interest statement in the “Confidential to Editor” section, and submit your "Accept" recommendation.

Reviewer #2: All comments have been addressed

Reviewer #3: All comments have been addressed

**********

2. Is the manuscript technically sound, and do the data support the conclusions?

The manuscript must describe a technically sound piece of scientific research with data that supports the conclusions. Experiments must have been conducted rigorously, with appropriate controls, replication, and sample sizes. The conclusions must be drawn appropriately based on the data presented.

Reviewer #2: Yes

Reviewer #3: Yes

**********

3. Has the statistical analysis been performed appropriately and rigorously?

Reviewer #2: Yes

Reviewer #3: Yes

**********

4. Have the authors made all data underlying the findings in their manuscript fully available?

The PLOS Data policy requires authors to make all data underlying the findings described in their manuscript fully available without restriction, with rare exception (please refer to the Data Availability Statement in the manuscript PDF file). The data should be provided as part of the manuscript or its supporting information, or deposited to a public repository. For example, in addition to summary statistics, the data points behind means, medians and variance measures should be available. If there are restrictions on publicly sharing data—e.g. participant privacy or use of data from a third party—those must be specified.

Reviewer #2: No

Reviewer #3: Yes

**********

5. Is the manuscript presented in an intelligible fashion and written in standard English?

PLOS ONE does not copyedit accepted manuscripts, so the language in submitted articles must be clear, correct, and unambiguous. Any typographical or grammatical errors should be corrected at revision, so please note any specific errors here.

Reviewer #2: Yes

Reviewer #3: Yes

**********

6. Review Comments to the Author

Please use the space provided to explain your answers to the questions above. You may also include additional comments for the author, including concerns about dual publication, research ethics, or publication ethics. (Please upload your review as an attachment if it exceeds 20,000 characters)

Reviewer #2: Dear authors, most of the comments are met and rechecked. However, the data repository was important and support the study.

Reviewer #3: Authors have adequately addressed all comments, and the manuscript should be accepted for publications.

**********

7. PLOS authors have the option to publish the peer review history of their article (what does this mean?). If published, this will include your full peer review and any attached files.

If you choose “no”, your identity will remain anonymous but your review may still be made public.

**Do you want your identity to be public for this peer review?**  For information about this choice, including consent withdrawal, please see our Privacy Policy.

Reviewer #2: **Yes:** yumna ali

Reviewer #3: No

**********

# Editor report: Acceptance letter

**DOI:** 10.1371/journal.pone.0351724.r006

**Authors:** Kshitij Karki

PONE-D-25-35396R2

PLOS One

Dear Dr. Palmieri,

I'm pleased to inform you that your manuscript has been deemed suitable for publication in PLOS One. Congratulations! Your manuscript is now being handed over to our production team.

At this stage, our production department will prepare your paper for publication. This includes ensuring the following:

* All references, tables, and figures are properly cited

* All relevant supporting information is included in the manuscript submission,

* There are no issues that prevent the paper from being properly typeset

You will receive further instructions from the production team, including instructions on how to review your proof when it is ready. Please keep in mind that we are working through a large volume of accepted articles, so please give us a few days to review your paper and let you know the next and final steps.

Lastly, if your institution or institutions have a press office, please let them know about your upcoming paper now to help maximize its impact. If they'll be preparing press materials, please inform our press team within the next 48 hours. Your manuscript will remain under strict press embargo until 2 pm Eastern Time on the date of publication. For more information, please contact onepress@plos.org.

You will receive an invoice from PLOS for your publication fee after your manuscript has reached the completed accept phase. If you receive an email requesting payment before acceptance or for any other service, this may be a phishing scheme. Learn how to identify phishing emails and protect your accounts at https://explore.plos.org/phishing.

If we can help with anything else, please email us at customercare@plos.org.

Thank you for submitting your work to PLOS ONE and supporting open access.

Kind regards,

PLOS ONE Editorial Office Staff

on behalf of

Dr. Kshitij Karki

Academic Editor

PLOS One
